# Mass Spectrometry Metabolomics and Feature-Based Molecular Networking Reveals Population-Specific Chemistry in Some Species of the *Sceletium* Genus

**DOI:** 10.3389/fnut.2022.819753

**Published:** 2022-03-29

**Authors:** Kaylan Reddy, Marietjie A. Stander, Gary I. Stafford, Nokwanda P. Makunga

**Affiliations:** ^1^Department of Botany and Zoology, Faculty of Natural Sciences, Stellenbosch University, Stellenbosch, South Africa; ^2^Department of Biochemistry, Faculty of Natural Sciences, Stellenbosch University, Stellenbosch, South Africa; ^3^Department of Plant and Soil Sciences, University of Pretoria, Pretoria, South Africa

**Keywords:** alkaloid chemistry, eco-metabolomics, kanna, kougoed, mesembrine, molecular networks

## Abstract

The *Sceletium* genus has been of medicinal importance in southern Africa for millennia and *Sceletium tortuosum* (Aizoaceae), one of eight species in the genus has gained pharmaceutical importance as an anxiolytic and anti-depressant due to the presence of mesembrine alkaloids. *S. tortuosum* is used for the manufacture of herbal teas, dietary supplements and other phytopharmaceutical products. This study aimed to provide a metabolomic characterization of *S. tortuosum* and its sister species as these are not easy to distinguish using morphology alone. Plant samples were thus collected from various locations in the succulent Karoo (South Africa) and analyzed through liquid chromatography-mass spectrometry (LC-MS), using MS^E^ fragmentation as a putative tool for chemical identities. Metabolomics-based analyses in combination with molecular networking were able to distinguish between the four species of *Sceletium* based on the presence of 4-(3,4-dimethyoxyphenyl)-4-[2-acetylmethlamino)ethyl]cyclohexanone (*m/z* 334.2020; RT 6.60 min), mesembrine (*m/z* 290.1757; RT 5.10 min) and 4'-O-demethylmesembrenol (*m/z* 276.1597; RT 4.17 min). Metabolomic profiles varied according to the different localities and metabolites occurred at variable quantitative levels in *Sceletium* ecotypes. Molecular networking provided the added advantage of being able to observe mesembrine alkaloid isomers and coeluting metabolites (from the joubertiamine group) that were difficult to discern without this application. By combining high-throughput metabolomics together with global and feature based-molecular networking, a powerful metabolite profiling platform that is able to discern chemical patterns within and between populations was established. These techniques were able to reveal chemotaxonomic relationships and allowed for the discovery of chemical markers that may be used as part of monitoring protocols during the manufacture of phytopharmaceutical and dietary products based on *Sceletium*.

## Introduction

*Sceletium tortuosum* (L.) N.E. Br (Aizoaceae syn. Mesembryanthemaceae), has well documented medicinal activity and ethnobotanical use as a psychoactive in southern Africa. Of the eight species in the genus, only *S. tortuosum* (commonly known as “kougoed”, “kanna” or “channa”) is used in the emergent phytopharmaceutics industries in South Africa and this use stems from the ethnobotany of the Khoi-San. The plant has been administered in a dried or fermented form for traditional uses ranging from pain and thirst relief to mood elevation (1). The mood-elevating activity of *S. tortuosum* has been attributed to the mesembrine alkaloids (particularly mesembrine and mesembrenone), acting as serotonin reuptake inhibitors (2), amongst other observed activities. The list of *Sceletium* natural products manufactured from raw plant material of *S. tortuosum* (3), that are in some cases collected from the wild as farming of this species is limited, is ever-growing. These products are sold as herbal teas, dietary supplements and other phytopharmaceutics. However, only some of these products have been scientifically investigated with the greatest quantity of information available for Zembrin® in terms of *in vitro* and *in vivo* pharmacological data (4, 5) and more recently clinical studies focusing on this particular product as an anxiolytic and anti-depressant phytopharmaceutical (6–9). In international markets, *Sceletium* products are classified as food supplements that are also sold in the complementary and alternative medicines sector. *S. tortuosum* is thus fast gaining interest as an alternative supplement to assist with depression and anxiety (10). New clinical evidence has also shown the ergogenic benefits in relation to better cognition to individuals that take a *S. tortuosum* supplement (11).

There is still much information missing in terms of comprehensive phytochemical profiling of *S. tortuosum* as only the mesembrine class of alkaloids have been studied despite the species having other alkaloid classes such as joubertiamine, tortuosamine and Sceletium A4 (12, 13). For these latter mentioned groups, there is a paucity of information in terms of their contribution to the pharmacological activity observed for S. *tortuosum*. Virtually no information is available with regards to the other taxa of *Sceletium* and the chemotaxonomic relationships between *S. tortuosum* and its sister species are largely unresolved. Metabolomic analyses have thus, the possibility to elucidate chemotaxonomic signatures that may provide novel chemical markers that could be used to distinguish biochemical relatedness across the genus. Thus far, metabolomic studies have mainly been applied to *S. tortuosum*. For example, Shikanga et al. (12) identified five distinct chemotypes based on the mesembrine alkaloid distributions using a gas chromatography-mass spectrometry-driven metabolomics approach (12). This study highlighted the intra-species differences that are present in selected *S. tortuosum* populations, but no other *Sceletium* species were studied in this work even though the genus has other species that also produce mesembrine alkaloids. The influence of these different chemotypes on pharmacological activity are not known but it is plausible that some populations are of greater medicinal importance based on their alkaloid profiles. More recently Zhao et al. (13), in a proton (^1^H) nuclear magnetic resonance (NMR) and ultra-performance liquid chromatography-mass spectrometry (UPLC-MS)-based metabolomics study, further supported intra-species differences in *S. tortuosum* between two regionally separated populations located in the Northern Cape and Western Cape in South Africa, with the total quantified alkaloid concentrations being higher in the Northern Cape plants (13).

**Figure 1:**
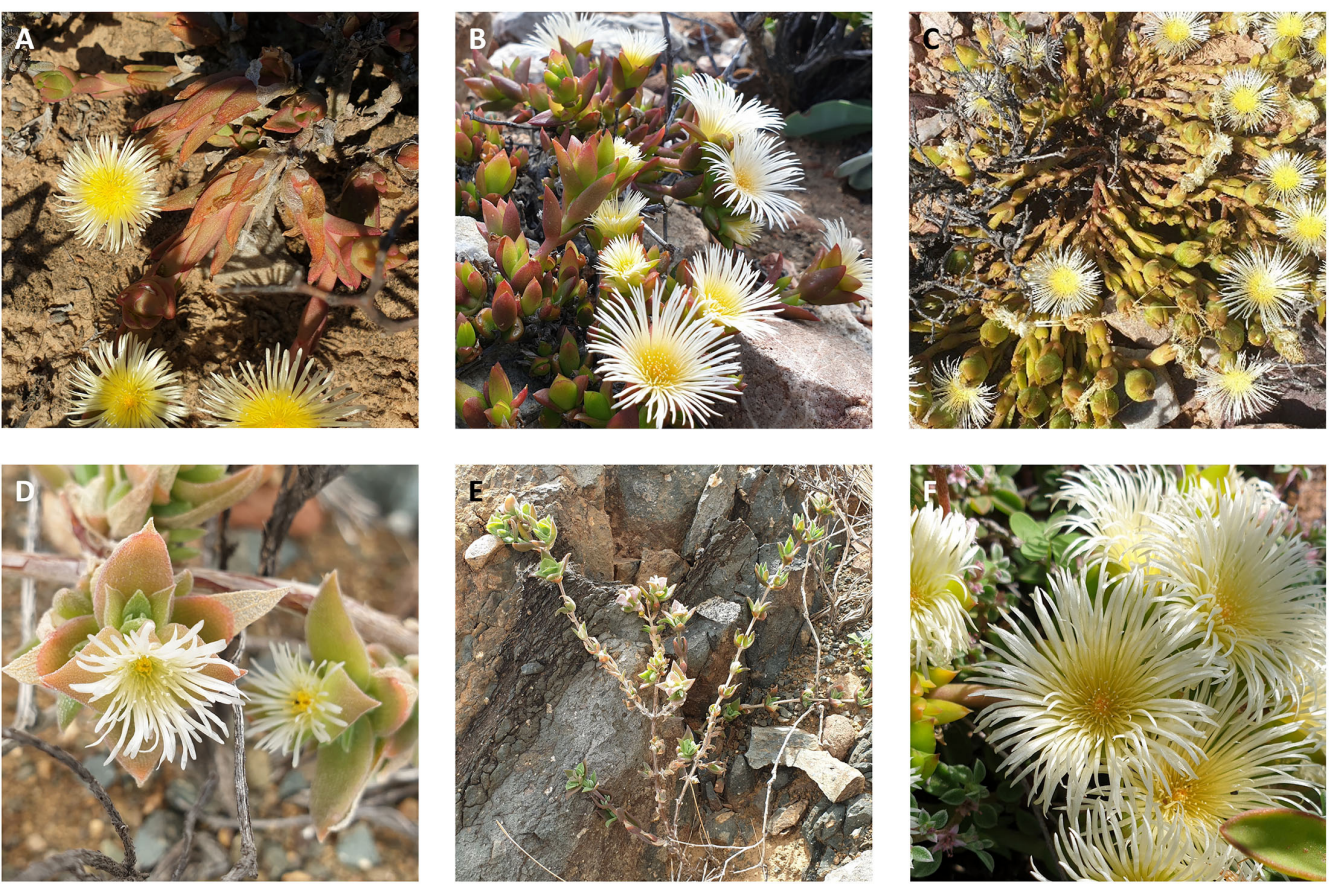
Species of *Sceletium* collected from the wild. **(A)** *Sceletium tortuosum* (De Rust); **(B)** *Sceletium strictum* (Anysberg); **(C)** *Sceletium emarcidum*; **(D)** *Sceletium rigidum*; **(E)** Upright growth-form of *Sceletium rigidum*; **(F)** *Sceletium tortuosum* (Ladismith 1).

It is apparent that *S. tortuosum* populations differ based both on geographic locations (13) and within the same region (12, 13), illustrating the high variability in chemistry across populations of *S. tortuosum* and the discriminatory power of using metabolomics to study medicinally important plants. This tool is gaining much popularity to pinpoint metabolite differences that may affect the quality of phytopharmaceuticals generated from particular populations. This paper aimed to contribute valuable insight into the metabolite differences between populations of *S. tortuosum* in the Western Cape and offer some chemotypic comparisons with closely related species in the genus, which have been largely absent until now, such as *S. rigidum*, L. Bolus., *S. emarcidum* (L.) L. Bolus, and *S. strictum* L. Bolus. Some of these are difficult to distinguish from *S. tortuosum* in terms of their morphological characters (Figure 1). The hypothesis for this paper was that there would be clear unique chemistry between different species of *Sceletium* and populations of the same species would be distinguishable based on their geographic locations. With this in mind, we chose to also explore molecular networking as a dereplication tool. Metabolomics paired with molecular networking may assist in distinguishing populations from each other with a great deal of phytochemical detail, especially where the chemistry is still largely unresolved (1). The feature identification capabilities of molecular networking act as a dereplication tool through identifying coeluting isobaric compounds and reducing redundancy by identifying isomers across samples from MS^2^ fragmentation patterns (14). This application of annotating the chemical space with less redundancy or misidentifications aids in vastly improving the separation power seen in downstream metabolomics analyses (14).

Molecular docking has also been utilized in this study to add *in silico* functional data to understand which phytochemicals may be responsible for the pharmacological activity observed *in vitro, in vivo*, and in clinical trials. This particular analysis, thus, aimed to elucidate if there are other minor phytochemical constituents from the *Sceletium* genus that may affect biological systems contributing to physiological responses in relation to anxiety and depression. Other species in the genus have been poorly explored in terms of their chemical diversity and pharmacological value even though they may also potentially assist with these neurological diseases. Using metabolomic tools in combination with molecular networking in this paper, it was possible to better define and characterize the extracts of plants of *Sceletium* occurring as wild populations and delineate qualitative and quantitative phytochemical species-specific differences. Tentatively identified metabolites were then assessed using molecular docking against pharmacological targets that modulate anxiety and depression. In this study, we not only investigate the chemical diversity within the genus but also draw predictions on which alkaloids of *Sceletium* are likely responsible for the neurological activity of this natural product.

## Methods and Materials

### Plant Collection and Glasshouse Cultivation

**Figure 2:**
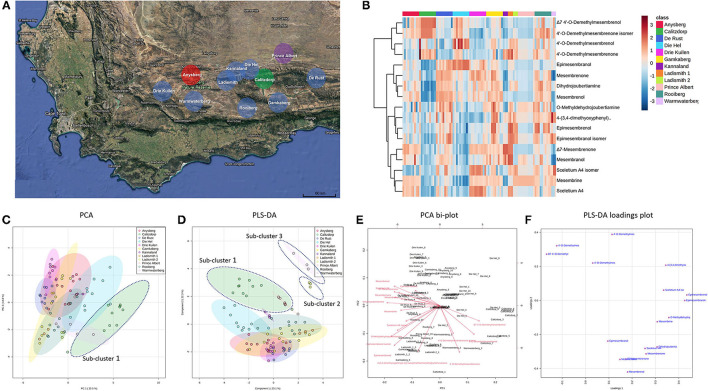
**(A)** Map showing geographic locations of *S. tortuosum* (blue), *S. strictum* (red), *S. rigidum* (purple) and *S. emarcidum* (green) found in South Africa; **(B)** Heat-map illustrating alkaloid diversity of tentatively identified alkaloids from all twelve *Sceletium* populations; **(C)** Principal component analysis (PCA) on all populations of *Sceletium*.; **(D)** Supervised partial least squares discriminant analysis (PLS-DA) on all populations of *Sceletium*; **(E)** PCA bi-plot of phytochemicals contributing to separation; **(F)** PLS-DA loadings plot of phytochemicals contributing to separation.

Specimens of *Sceletium sp*., were collected from various localities in the Western Cape (*n* = 12) in South Africa at the end of the winter season in August-September of 2020 (Table 1; Figure 1). Geographic locations can be seen in Figure 2A. Voucher specimens were lodged at the Stellenbosch University Herbarium and a collection of plants was established in the glasshouse and the Stellenbosch University botanical garden. The taxonomic identities of these species were confirmed by Dr. D. Kirkwoord and the *S. rigidum* plants were collected with Dr. S. Dean, a trained plant scientist.

**Table 1:** Species, population localities, and photoperiods of samples investigated in this study.

**Species**	**Population locality**	**Photoperiod**	**Voucher number**
*Sceletium tortuosum*	Warmwaterberg	12 h 20 min	Reddy 001 SU
	Rooiberg	12 h 16 min	Reddy 002 SU
	Gamkaberg	10 h 34 min	Reddy 003 SU
	Drie Kuilen	12 h 5 min	Reddy 004 SU
	Die Hel	10 h 34 min	Reddy 005 SU
	De Rust	10 h 35 min	Reddy 006 SU
	Kannaland	12 h 15 min	Reddy 007 SU
	Ladismith 1	12 h 15 min	Reddy 008 SU
	Ladismith 2	12 h 15 min	Reddy 009 SU
*Sceletium strictum*	Anysberg	12 h 8 min	Reddy 010 SU
*Sceletium emarcidum*	Calitzdorp	12 h 13 min	Reddy 011 SU
*Sceletium rigidum*	Prince Albert	12 h 11 min	Reddy 012 SU

### Overview of Collection Sites

Plants were sampled from 12 sites throughout the Western Cape Province of South Africa (Table 1; Figure 2A). The plants from De Rust grew in the Riversdale bioregion (Figure 1A) and were well established with a robust habit, even though in most cases, were growing under other shrubs with large portions exposed to the sun. At the time of collection, these plants were exposed to sunlight for a period of 10 h 35 min (Table 1). These plants were red in colour, most likely containing betalains which are known to occur in plants from the Caryophyllales such as *Sceletium* (15). The plants from Anysberg fall within the Moordenaars Karoo, Gouritz and Witteberg bioregions (Figure 1B) and this population was growing in very rocky slopes of a steep hill in a dry environment, fully exposed to sunlight. Similarly, to those from the De Rust area, Anysberg plants were also showing a red colouration. Calitzdorp occurs in the Sandveld bioregion (Figure 1C) and during the time of collection sunrise and sunset in Calitzdorp was from 06:36 to 18:23, respectively, and *S. emarcidum* harvested from this region were approximately 30 cm, in most cases. *S. rigidum* was obtained from Prince Albert (Swartberg bioregion) and grew as a highly restricted population toward the top of a hill (Figure 1D) and plants were exposed to 12 h 11 min of light. This species grew against, or very close, to large boulders where the soil was very hard (Figure 1E), in an upright fashion. The area of Ladismith (Gouritz bioregion) has quartz rocks and two collections of *S. tortuosum* were made in this region and the area is referred to as the Ladismith 2 location (Figure 1F). These plants were growing on a south facing slope with quartz and shale rich soil. Samples collected from Warmwaterberg were in *in situ* conditions characteristic of The Koo bioregion. The plants harvested from this site were small (10 cm in diameter) and fully exposed to the sun as the region had been through a drought with many other small shrubs that could provide canopy cover to *S. tortuosum* appearing to be completely desiccated and bare. Although many other localities were dry with little to no rainfall in September, the Drie Kuilen (The Koo bioregion) population had been exposed to mist and the leaves of the *S. tortuosum* were a vibrant green, growing to about 15–20 cm in diameter. Most of these plants grew quite protected underneath the canopy of other established shrubs and bushes that are native to the area and the daylength was 12 h 5 min. The plants from Kannaland (Gouritz and Sandveld bioregions) were healthy and growing under other shrubs and plants. Sunrise and sunset in Kannaland were from 06:38 to 18:23. Some were pigmented with a red color, but others were growing in fairly shaded environments and were greener in their appearance. These plants occurred in the Gouritz and Sandveld bioregions. The Die Hel population falls in the Sandveld bioregion and were growing in dry soil that had many rocks. The plants grew on a very steep slope where they were exposed, showing signs of vining down the rocky slopes. The Rooiberg population forms part of the Sandveld bioregion. These plants grew mainly protected by larger shrubs and were well established (10–20 cm in diameter). The Gamkaberg population occurred within the Sandveld bioregion. Sunrise and sunset in Gamkaberg were from 07:20 to 17:54 (Table 1). This population had many individuals that were red to maroon in color as this area is likely to have high light intensities and UV exposures that these plants throughout the year (16–18). The plants grew in very rocky (shale) soil where the ground was compact. This population grew across a slight south facing incline. The region has experienced an intense drought when samples were collected.

### Phytochemical Extraction

Four different species of *Sceletium* (Table 1) were dried in silica in a sealed plastic bag, in darkness at room temperature. Leaves were ground to a fine powder using liquid nitrogen using a mortar and pestle. For each extraction, the sample powder was weighed and transferred into a 2 ml Eppendorf® Safe-Lock microcentrifuge polypropylene tubes. Samples were extracted using methanol as an extraction solvent, maintaining a concentration of 50 mg/mL of plant sample to solvent. Samples were vortexed (20 s), sonicated (20 min), (Branson 50/60 Hz, Branson Cleaning Equipment Company, USA) and benchtop centrifuged (10 min) (Hermle Z160m, 3,000 x g). The supernatant was aliquoted (1 ml) into autosampler vials for metabolite analysis. A total of 107 samples were analyzed. All samples were stored at 5°C and analyzed within 24 h of being extracted.

### Ultra-High Performance Liquid Chromatography-Mass Spectrometry

Chemical analysis was executed on an Acquity ultra-high-performance liquid chromatography (UHPLC) system (Waters Corporation, USA) coupled to a Waters Acquity photodiode array (PDA) UV detector (230–500 nm) and Synapt G2 HDMS qToF mass spectrometer (Waters Corporation, USA). Separation of metabolites was performed on a UPLC™ BEH C_18_ column (2.1 x 100 mm, i.d., 1.7μm particle size, Waters). Electrospray ionization was applied in the positive mode (ESI+) using a Z-spray source with the following ionization conditions; 15 V cone voltage, 2.5 kV capillary voltage, 120°C source temperature, 50 L/hr cone gas flow. Nitrogen at 650 L/hr was used as the desolvation gas and a desolvation temperature of 275 °C was applied. A Water Acquity UPLC Binary Solvent Manager delivered the mobile phase solvents at a flow rate of 0.4 ml/min. The gradient was initiated at 90% 0.1% ammonium hydroxide in water (Solvent A) and held for 30 s, followed by a linear gradient transition to 100% acetonitrile containing 0.1% ammonium hydroxide (Solvent B) over 9.5 min, followed by a return to 90% Solvent A over 0.1 min, remaining here for 2.9 min to re-equilibrate the column and giving a total run time of 13 min. Ammonium hydroxide was used as a mobile phase as it was found to induce the best separation of *Sceletium*-derived alkaloids when the method was being developed. At high pH, the alkaloids possess a neutral charge, and this gave better chromatographic peak shapes and retention times. However, a low pH results in the alkaloids being positively charged and are thus not well retained on the reverse phase system used in this study. Mass spectral data were attained using an 160–1,500 Da range window. Data were centroided during acquisition and the LockSpray™ module was used to ensure mass accuracy with leucine encephalin as reference. A 3 μl sample injection volume was used. Methanol (HPLC grade; UV cut-off 215 nm) and acetonitrile (UV cut-off 200 nm) (ROMIL Ltd., Microsep, South Africa) were used for sample preparation and for the mobile phase. Dilution of reagents in all cases used, ultrapure analytical grade Type 1 water (Milli-Q®, Merck, Darmstadt, Germany).

### Data Interpretation and Analysis

#### Quantitative Chemical Analysis

Two independent LC-MS analyses were conducted, and the datasets were combined irrespective of the LC-MS run. From the combined dataset, feature picking was conducted manually with 18 different features that occurred between retention time 3.00 min and retention time 7.00 min. The selection of metabolites was initially based on published literature and the metabolomic analyses (described in Section Multivariate Statistical Analysis: PCA and PLS-DA) were also used to guide the selection of chemical markers. These 18 features were selected based on literature sources where chemicals where identified in *Sceletium* species (order of elution and MS/MS fragmentation patterns), Variable Importance in Projection (VIP) scores that indicate those metabolites contributing the greatest differences amongst populations, bi-plots and loadings plots across metabolomic experiments, chemical clusters identified from spectra deconvolution in molecular networking, feature picking in MS-DIAL, as well as formula identification from elemental composition analysis based on MS1 spectra in MassLynx. Data acquisition and processing were carried out using TargetLynx™ Application Manager for MassLynx™ v4.1 software (Waters Corporation, USA) for quantitation (mg/kg DW) of compounds using the integrated peak areas of extracted mass chromatograms and mesembrine as the in-house reference standard. A *m/z* range of 262 to 334 and an average threshold of 1.5 min was used for the quantification of phytochemicals. Each peak was inspected, and peak areas selected manually where errors were observed in TargetLynx. Relative quantification was carried out using a concentration range of the mesembrine standard from 3.125 to 100 ppm where each metabolite quantified according to this straight-line curve where volume and mass used in the extraction was also taken into account. Phytochemicals were quantified and reported as dry weight (DW) yield (mg/kg). The numerous putatively identified alkaloid metabolites of interest in samples, for which no commercial standards exist, were quantified relative to the mesembrine standard, and expressed as mesembrine equivalents, facilitating their semi-quantitative analysis and comparison. Alkaloid identification was conducted by referencing the experimental *m/z*, elution time, UV spectra, and elemental composition analysis (±5 ppm tolerance) to published structures, in-house reference standards data sets (mesembrenone and mesembrenol), and where possible confirmed by MS^E^ fragmentation-PDA datasets. Chemical structures of compounds and molecular details can be found in Supplementary Material A and fragmentation spectra and full chromatograms are indicated in Supplementary Material B.

#### Multivariate Statistical Analysis: PCA and PLS-DA

The multivariate data analysis tool of a principal component analysis (PCA) and partial least squares discriminant analysis (PLS-DA) of the UHPLC-MS data set was used to visualize qualitative differences in chemical composition of different populations and species of *Sceletium*. Detection of relevant chemical marker peaks and the generation of the data matrix from the large, raw UHPLC-MS chromatographic datasets was performed in MS-DIAL (v4.70). In MS-DIAL, the parameters of data collection were set to a retention time window of 1.5 to 8.5 min, an MS1 mass range of 120 to 1,200 Da and an MS/MS mass range of 40 to 1,000 Da. Peak detection was set to an amplitude of 1,000 and a retention time tolerance of 0.05 min. The resulting pre-processed dataset matrix was exported to an excel workbook format (.xlsx) and then converted to a comma delimited file format (.csv) that was then analyzed using MetaboAnalyst (http://metaboanalyst.ca). Peak intensity data were filtered using median intensity to remove variables that were unlikely to be of use when modeling the data. Sample normalization was done by sum, to adjust for systematic differences among samples. Data were log transformed and auto scaled (mean-centered and divided by the standard deviation of each variable). Score plots and loading plots were further used to identify key metabolites contributing to the differences between and within populations (Table 2). These metabolites were then tentatively identified based on the MSE fragmentation patterns, literature sources, molecular masses and predicted molecular formulae. The selection of metabolites was then used in further metabolomic experiments. Heatmaps with hierarchal cluster analyses (HCA) were also created from the phytochemicals found in plant populations, and the study species. Model validation with R2, Q2 and accuracy scores were conducted using MetaboAnalyst, and are reported in Supplementary Material C. Classification and cross-validation were performed in MetaboAnalyst using the wrapper function in the caret package (21) which was ran using R-code on the MetaboAnalyst website.

**Table 2:** Tentatively identified alkaloids from *Sceletium* species collected from the wild.

**Network number**	**Compound name**	**Alkaloid chemical class**	***m/z* (M+H^**1**^)**	**Retention time (min)**	**Molecular formula**
1	Mesembrine^d^	Mesembrine	290.1757	5.10	C17H24NO_3_
3	Mesembranol^d^	Mesembrine	292.1913	4.61	C17H26NO_3_
	Δ7-Mesembrenone^a,b,c^	Mesembrine	288.1600	4.53	C17H22NO_3_
	Mesembrenone^a,b,c^	Mesembrine	288.1596	4.82	C17H22NO_3_
4	Mesembrenol^d^	Mesembrine	290.1763	4.54	C17H24NO_3_
	O-Methyldehydrojoubertiamine^a^	Joubertiamine	272.1674	4.54	C17H22NO_2_
7	Sceletium A4^a,b,c^	Sceletium A4	325.1914	5.47	C20H25N2O_2_
	Sceletium A4 isomer^a,b,c^	Sceletium A4	325.1910	5.69	C20H25N2O_2_
8	6-Epimesembranol / Epimesembranol^a^	Mesembrine	292.1884	6.57	C17H26NO_3_
10	4'-O-Demethylmesembrenone^a^	Mesembrine	274.1442	3.89	C16H19NO_3_
11	6-Epimesembrenol / Epimesembrenol^a^	Mesembrine	290.1748	5.35	C17H24NO_3_
12	Dihydrojoubertiamine^a^	Joubertiamine	262.1805	5.47	C16H24NO_2_
13	4'-O-Demethylmesembrenone isomer^a,b^	Mesembrine	274.1446	4.11	C16H19NO_3_
14	4-(3,4-dimethyoxyphenyl)-4-[2-acetylmethlamino)ethyl] cyclohexanone^a^	Joubertiamine	334.2020	6.60	C19H28NO_4_
15	O-Acetylmesembrenol^a^	Mesembrine	332.1875	5.98	C19H26NO_4_
16	4'-O-Demethylmesembrenol^a,b^	Mesembrine	276.1597	4.17	C16H22NO_3_
18	Δ7-4'-O-Demethylmesembrenol^a,b^	Mesembrine	276.1602	4.09	C16H22NO_3_

*Tentative identifications proposed according to structures and exact masses published by Gericke and Viljoen (1)^a^; Patnala and Kanfer (19)^b^ and Roscher et al. (20)^c^; ^d^Identification confirmed with a purified chemical standard*.

With regards to the metabolomic analyses, there were three separate experiments conducted:

1. Metabolomic analyses on the populations irrespective of species delineations were analyzed together, to assess the metabolomic trends across all the populations.
2. A comparison of the metabolomic differences where *S. tortuosum* occurred was conducted to determine intra-species metabolite trends based on locality; and,
3. *S. strictum, S. rigidum, S. emarcidum* and *S. tortuosum* were analyzed together to provide a context of the metabolite differences amongst these four species.

#### Univariate Statistical Analyses

A one-way analysis of variance (ANOVA) for all quantitative data were performed using GraphPad Prism version 8.0.1 for Windows (GraphPad Software, San Diego, California USA (www.graphpad.com). Prior to this, D'Agnostino-Pearson omnibus normality test was conducted to test if the data conformed to a normal (Gaussian) distribution. A total of 10 replicates was used in most analyses. For the Kannaland, Ladismith, and the Warmwaterberg populations, only three replicates could be used. Data were also manually assessed for outliers and where present these were removed on a case-by-case basis. To separate the means, multiple comparisons were conducted as a *post-hoc* test, for pairwise comparisons. A *post-hoc* Tukey test was thus used to test the statistical hypothesis for normally distributed data. In cases where data did not conform to assumptions of normality, a non-parametric test using Kruskal-Wallis analysis was regarded as being most appropriate. Descriptive statistics using boxplots or stacked column charts were also employed to visualize the data.

### Feature-Based Molecular Networking

Raw data files were converted to mzML files using the MS convert tool (ProteoWizard version 3.0.1904) and loaded onto MS-DIAL where data from SWATH-MS^2^ (data independent LC- MS^2^ acquisition) was employed. Retention time tolerance and MS1 tolerance were set to 0.1 min and 0.02 Da, respectively. Data from the UHPLC-MS^E^ analysis of *Sceletium* populations was processed in MS-Dial to perform feature picking. This process enabled the identification of minor chemical constituents as well as co-eluting compounds. Once peak detection and MS^E^ deconvolution was complete the MS^E^ spectra were exported as a mgf file and qualitative data as a csv file which were both used to construct molecular networks on the Global Natural Products Social Molecular Networking (GNPS) platform (https://gnps.ucsd.edu). Parameters for feature-based molecular networking were set as follows: precursor ion mass tolerance, 0.05; min pair cosine, 0.7; network topK, 10; maximum connected component size, 100; minimum matched product ions, 3; minimum cluster size, 6. The feature-based molecular network was constructed and visualized in CytoScape (version 3.7.0). Once data were opened in CytoScape, maps were altered with regards to styles of nodes, Edge and Network. Nodes were annotated and scaled with respect to parent ion masses. Distance between nodes was set to represent MS2 spectrum similarity. Networks were overlaid with geographic location and visualized with Passthrough mapping settings. Label colors, position and size were altered for best visual appearance. For retention time networks, nodes were colored by retention time using Passthrough mapping.

### Molecular Docking Using Glide

An approach of using metabolomic tools to tentatively identify key metabolites in *Sceletium* was used initially to generate a list of molecules that may be responsible for the mood elevation activity of *Sceletium* (2, 22). These molecules could then be tested *in silico* against a number of receptors that assist in the modulation of Alzheimer's, Parkinson's, anxiety and depression. This was regarded as being important to enable the identification of populations or species that may hold neurological activity aside from those previously studied as well as provide insight into the receptor binding capacities of the chemical markers that were tentatively identified using molecular networking and the metabolomics methods.

#### Dataset Collection

The ligand dataset was prepared from molecules that were tentatively identified in a previous portion of this study (detailed previously in Section Multivariate Statistical Analysis: PCA and PLS-DA) using the LigPrep algorithm in Schrodinger Maestro. These 18 molecules belong to the following chemical groups: mesembrine alkaloids, joubertiamine alkaloids and Sceletium A4 alkaloids (Table 2). The structures of the compounds were collected from the PubChem database for further analysis and when not available, were constructed in ChemDraw (Supplementary Material A). The approach of using tentatively identified metabolites was used to place the metabolite diversity present in *Sceletium* species in context of the therapeutic use of *Sceletium* as a mood elevator. Taking this approach could enable us to identify metabolites that may not occur in high concentrations but may hold therapeutic activity. The proteins that were investigate for *in silico* screening were the 5-HT serotonin transporter (5I75) (23), the GABA-A receptor (6D6T) (24), and the acetylcholinesterase (AChE) enzyme (1QTI) (25). These proteins and enzymes were selected on the basis that they respond best to mesembrine alkaloids in a wide screening of a receptor binding assay performed by Harvey et al. (22). The acetylcholinesterase enzyme was selected on the basis of previously reported activity of *Sceletium* to assist with cognitive enhancement (Alzheimer's) (7, 26, 27).

#### Preparation of Protein and Ligands

The crystal structures of the receptors of interest were imported from the Protein Data Bank (PDB) and were downloaded in pdb format and prepared for the docking process. The proteins were prepared using the Protein Preparation Wizard in Maestro (28), using standard settings. Alterations to hetero-groups and water removal was performed once the protein was prepared (29) in the Schrodinger protein preparation suite (30). The experiments were performed at pH ranges (pH 7.4 +/-2) simulating that of biological ranges. Ligands were also prepared into a conformation that is energetically favorable for molecular docking. The ligands were prepared using the LigPrep Schrodinger suite (30).

#### Molecular Docking of Compounds and Model Validation

The ligand docking was performed using glide in Maestro (29) of the Schrodinger suite (30) with the default settings (sample nitrogen inversions, sample ring conformations and add Epik state penalties to docking score) with Extra Precision (XP). The receptor grid size was appropriately selected based on the amino acid distribution in the binding pocket. In all three binding sites the receptor grid size was found to be most optimal at 6 Å. Results of model validation can be found in Supplementary Material D.

Model validation was performed to analyse if the model was possible and accurately simulates biological environment. This was performed by comparing the docked compound to a reference compound and positive control to see the RMSD and binding activity relative to a positive control. A RMSD value of 1.5 Ångström or smaller was be considered an appropriate model simulation (see Supplementary Material D).

## Results and Discussion

### Chemical Profiling of *Sceletium* Populations

**Figure 3:**
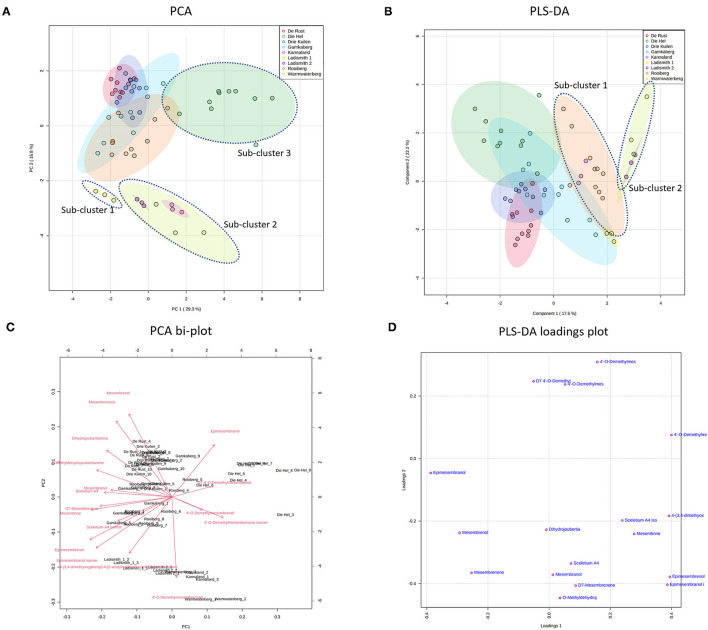
**(A)** PCA on all populations of *S. tortuosum* used in the study. **(B)** PLS-DA on all populations of *S. tortuosum*; **(C)** PCA bi-plot of phytochemicals contributing to separation between populations of *S. tortuosum*; **(D)** PLS-DA loadings plot of phytochemicals contributing to the separation.

**Figure 4:**
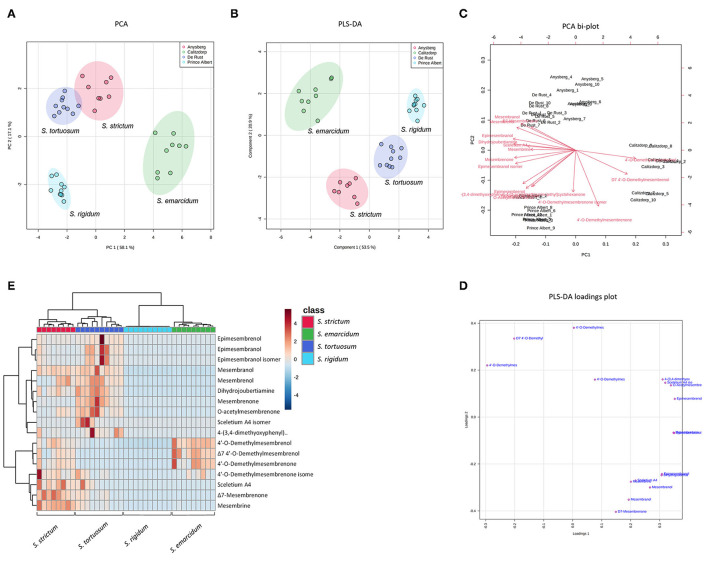
**(A)** PCA on four species of *Sceletium*. (*S. rigidum; S. emarcidum; S. tortuosum* and *S. strictum*); **(B)** Supervised PLS-DA on four species of *Sceletium*; **(C)** PCA bi-plot of phytochemicals contributing to separation among four species of *Sceletium*; **(D)** PLS-DA loadings plot of phytochemicals contributing to the separation; **(E)** Heat map of phytochemical distribution among four *Sceletium* species generated using selected chemical markers.

**Figure 5:**
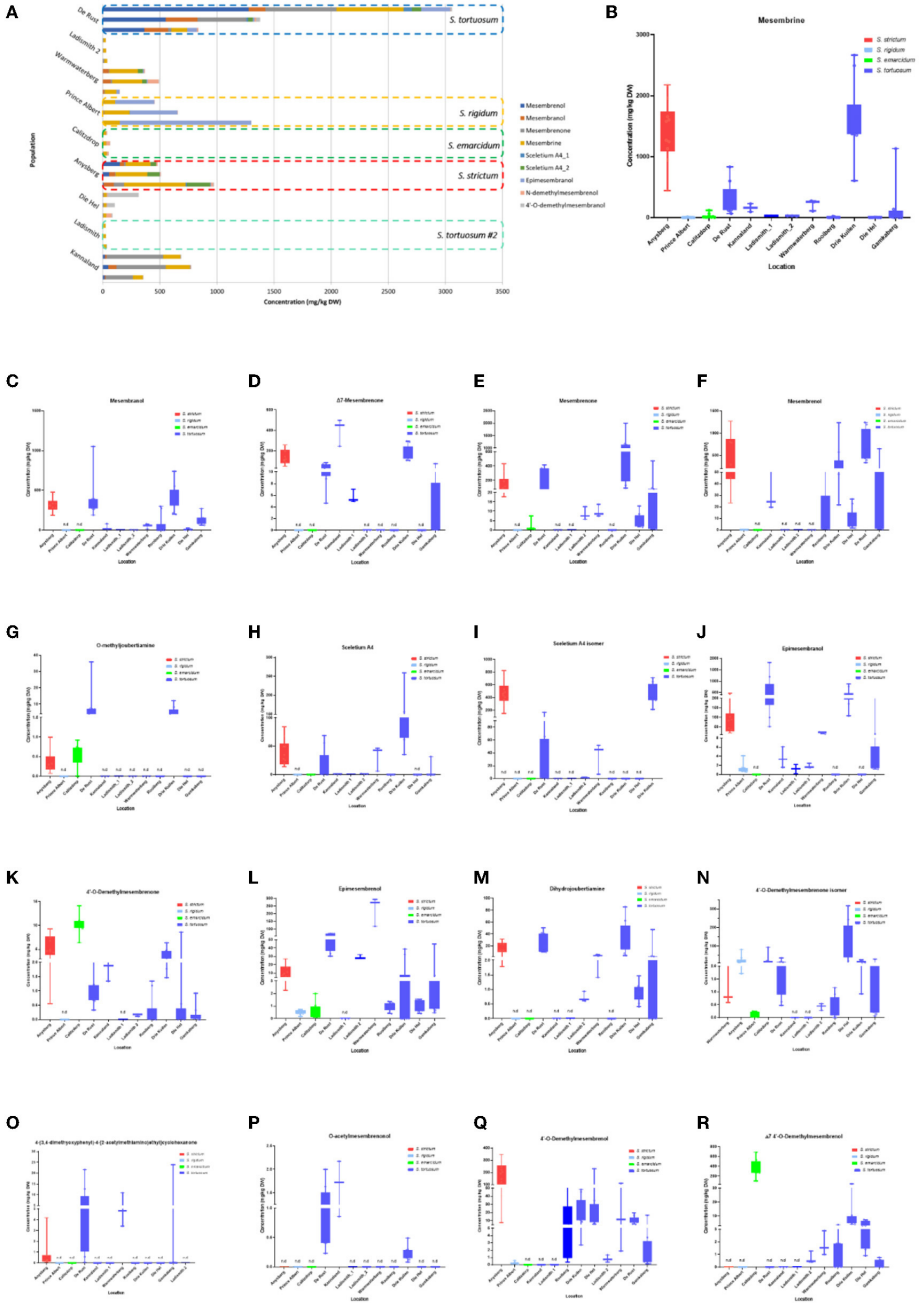
**(A)** Concentration of *Sceletium* metabolites in different species and populations found at twelve locations. Horizontal stacked columns represent metabolites (mg/kg DW) tentatively identified chemical markers responsible for metabolomic separations. Each horizontal stacked column represents up to 10 individuals per location and/or taxon *S. tortuosum* B-R) Concentration of tentatively identified metabolites (mg/kg DW) in *S. strictum, S. emarcidum, S. tortuosum* and *S. emarcidum* found at different locations: **(B)** Mesembrine; **(C)** Mesembranol; **(D)** Δ7-Mesembrenone; **(E)** Mesembrenone; **(F)** Mesembrenol; **(G)** O-methyldehydrojoubertiamine; **(H)** Sceletium A4; **(I)** Sceletium A4 isomer; **(J)** Epimesembranol; **(K)** 4'-O-Demethylmesembrenone; **(L)** Epimesembrenol; **(M)** Dihydrojoubertiamine; **(N)** 4'-O-Demethylmesembrenone isomer; **(O)** 4-(3,4-dimethyoxyphenyl)-4-[2-acetylmethlamino)ethyl]cyclohexanone; **(P)** O-acetylmesembrenol; **(Q)** 4'-O-demethylmesembrenol; **(R)** Δ7 4'-O-Demethylmesembrenol.

Methanolic extracts were analyzed in a LC-QToF-MS system prior to use of a semi-supervised metabolomic analysis. Metabolomic analyses were conducted to compare all populations of *Sceletium* (Figure 2), populations of *S. tortuosum* (Figure 3) and four species of *Sceletium* (Figure 4). Heat maps generated from the overall populations of *Sceletium* illustrated the relative alkaloid diversity within the twelve *Sceletium* populations (Figure 2B). Δ7 4′-O-demethylmesembrenol (Figure 5Q) and 4′-O-demethylmesembrenone isomer (Figure 5J) were present and occurred at the highest concentration in *S. emarcidum* collected from Calitzdorp. High levels of Δ7-mesembrenone and mesembrenol were present in the Kannaland and Ladismith 1 populations of *S. tortuosum*. The heat maps of all populations also illustrated a relatively high distribution of sceletium A4 in the Drie Kuilen population. An unsupervised (PCA) and supervised analysis (PLS-DA) of the metabolomic profile of these populations illustrated a considerable amount of overlap between the 12 populations (Figures 2C,D) where it was difficult to separate plants from a taxon-specific level. All samples were collected from the Western Cape province. No clear separation in samples collected from the Western Cape was initially observed (Figure 2C). Zhao et al. (13) also observed chemical differences in populations collected from the Western and Northern Cape provinces in South Africa suggesting province-specific metabolite profiles. Despite this, some groupings are discernable. For example, the plants from Die Hel, Calitzdorp and Warmwaterberg appeared to have chemical signatures that placed them on the positive side of PC1 (Figure 2C). The PCA analysis illustrated the separation of a subcluster from the Calitzdorp population (Figure 2C). The distinct formation of three separate sub-clusters, being from Calitzdorp, Ladismith 2 and Warmwaterberg (Figure 2D) was apparent with the PLS-DA. This analysis gave poor separation between samples, and this is suspected to be due to the great deal of shared chemistry between the locations tested. The metabolites causing the separation were tentatively identified as dihydrojoubertiamine, 4-(3,4-dimethyoxyphenyl)-4-[2- acetylmethlamino)ethyl]cyclohexanone, mesembrenol and 4'-O-demethylmesembre nol for the PCA (Figure 5). These metabolites, specifically dihydrojoubertiamine, 4-(3,4-dimethyoxyphenyl) 4-[2-acetylmethlamino)ethyl]cyclohexanone, mesembrenol and 4'-O-demethylmesembrenol, were highest in Drie Kuilen (34.63 ± 25.86 mg/kg DW) (Figure 5M), Warmwaterberg (6.359 ± 3.964 mg/kg DW; *p* < 0.0001) (Figure 5O), De Rust (2.795 ± 0.2072 mg/kg DW) (Figure 5F) and Anysberg (181.2 ± 105.4 mg/kg DW) (Figure 5Q), respectively. With respect to the PLS-DA loadings plot (Figure 2F), the greatest contributing metabolites to the separation of sub-cluster 3 and 2 (Figure 2D) was 4-(3,4-dimethyoxyphenyl)-4-[2- acetylmethlamino)ethyl]cyclohexanone and the Sceletium A4 isomer. The PLS-DA model showed an accuracy of 0.44565, R2 of 0.5529 and a Q2 score of 0.4000 for three components in the analysis.

The intra-population chemical differences in *S. tortuosum*, which was the most abundant species in terms of distribution are shown in Figure 3. A PCA performed on the data illustrated some separation but an overall overlap of populations. The populations of Die Hel, Warmwaterberg and Kannaland grouped on the positive side of PC1 whilst Kannaland, Ladismith 1 and 2 as well as Warmwaterberg are located on the negative side of PC2 (Figure 3A). Three sub-clusters were apparent and these are linked to the Ladismith 1, Warmwaterberg and Die Hel plants (Figure 3A). A PLS-DA analysis of intra-population differences, showed sub-clustering of Rooiberg and Warmwaterberg (Figure 3B). A bi-plot of the PCA (Figure 3C) indicated that the major contributors to separation of the Kannaland population was mesembrenol (1.480 ± 0.2515 mg/kg DW; Figure 5F) and Δ7-mesembrenone (397.8 ± 133.9 mg/kg DW; Figure 5D) and these were statistically significant (*p* < 0.0001). A large degree of variation was observed in the Die Hel population, this population was also sampled along a steep incline where some plants were exposed to full sun conditions whilst some were covered by other large plants. Influences on plant metabolites by light exposure have been observed in *Pteridium arachnoideum, Arabidopsis thaliana* and *Artemisia annua* among other plants (31–33). Light is essential in the regulation of developmental plasticity of plants and their secondary metabolites (34).

The production of specialized metabolites in plants is highly influenced by both biotic and abiotic factors and differences in soil composition were apparent for the various locations where plants of *Sceletium* were found. For example, the soil composition was also quite variable in the Die Hel site. Some plants grew in soft soil whilst others were growing in very shale rich soils. The nutrient composition of soil (rich organic matter, low pH, high levels of exchangeable nitrogen and total nitrogen) was a contributing factor to increase salidroside production, a specialized metabolite found in the Chinese medicinal herb, *Rhodiola sachalinensis* (35). In *Ceratonia siliqua*, high nutrient soil led to decreases in total leaf phenolics and tannins (36). With reference to the PLS-DA loadings plot (Figure 3D), the separation of sub-cluster 1 and 2 was linked to 4'-O-demethylmesembrenone and Δ7 4'-O-demethylmesembrenol. The PLS-DA model was recorded to have an accuracy of 0.56452 with the R2 value of 0.8378. A Q2 score of 0.7678 for three components in the analysis was evident.

A more supervised metabolomic experimental structure was used to reduce the redundancy of the metabolite data and assess the intra-population variation between the four *Sceletium* species (*S. rigidum; S. emarcidum; S. tortuosum* and *S. strictum*) and clear separations for both the PCA and PLS-DA plots were noted (Figure 4). To account for influences on phytochemistry based on growth location, samples were collected along inclines. Others have noted such effects where geographic coordinates may influence phenotypic plasticity of a population of plants with individuals expressing qualitative differences that are detected as intra-specific variation within a set of plants from this same species (37–42). *S. strictum* has a greatly restricted distribution as isolated community assemblages and it is interesting to note the high level of variability observed in alkaloid content in this particular species. Many factors are known to alter metabolite biosynthesis. For example, temperature was identified as being key in phenolic variation in *Arnica montana* (43). Similarly, cold acclimation altered the production of secondary metabolites thereby influencing the antioxidant capacity of *Petunia* × *hybrida* (44). In *Achnatherum inebrians*, the presence of salt and drought stress was a major influence on alkaloid production (45).

Figures 4A,B illustrate the PCA and PLS-DA analyses performed between the four *Sceletium* species, respectively. There is a clear delineation of all four species from each other. The PLS-DA model showed an accuracy of 1.0, R2 of 0.9783 and a Q2 score of 0.9355 for 5 components in the analysis. Based on the bi-plot, the metabolites largely influencing the PCA groups were the joubertiamine alkaloid, 4-(3,4-dimethyoxyphenyl)-4-[2- acetylmethlamino)ethyl]cyclohexanone, mesembrine and 4'-O-demethylmesembrenol (Figure 4C). The PLS-DA loadings plot showed the greatest contributing metabolites to the separation of species being Δ7 4'-O-demethylmesembrenol, 4'-O-demethylmesembrenol and 4-(3,4-dimethyoxyphenyl)-4-[2- acetylmethlamino)ethyl]cyclohexanone (Figure 4D). The heatmap provided further validation indicating that these alkaloids were in higher relative abundance in the plants collected from De Rust (*S. tortuosum*), Anysberg (*S. strictum*) and Calitzdorp (*S. emarcidum*), respectively (Figure 4E).

Eighteen different alkaloids were tentatively identified using MS^E^ fragmentation patterns, relative retentions times and accurate mass spectra and several of these metabolites were quantitatively higher in some of the populations, namely, Kannaland (*S. tortuosum*) and Ladismith 1 exhibiting higher amounts of Δ7-mesembrenone (m/z 288.1600) concentrations of 397.8 ± 133.9 mg/kg DW (*p* < 0.0001). Mesembrine (m/z of 290.1757), that is used as a chemical marker in manufactured products of *S. tortuosum* (1, 10), was highest in the plants collected from Drie Kuilen (1,640 ± 582.3 mg/kg DW) and Anysberg (1,402 ± 504.8 mg/kg DW). Sceletium A4 (m/z of 325.1914) that is structurally different from mesembrine by having a 2,3- disubstituted pyridine moiety and 2 nitrogen atoms, occurred in highest relative ion intensity in those plants that were collected from Drie Kuilen (114.5 ± 63.98 mg/kg DW; *p* < 0.0001).

Joubertiamine alkaloids had a higher distribution in *S. tortuosum* species collected from Warmwaterberg and De Rust. The joubertiamine alkaloid 4-(3,4-dimethyoxyphenyl) 4-[2-acetylmethlamino)ethyl]cyclohexanone was found in concentrations of 6.359 ± 3.964 mg/kg DW and 5.533 ± 6.848 mg/kg DW (Figure 5O), respectively in these populations. *S. rigidum* (Prince Albert) had considerably lower levels of alkaloids than the other species. In the study of Patnala and Kanfer (46) samples of *S. rigidum* were reported to not have any mesembrine alkaloids. This particular species is morphologically different from all the other species in the genus (Figures 1D,E) as it has an upright form with many prominent idioblasts and a highly restricted distribution. In this study, it could easily be distinguished from the other *Sceletium* collections due to the absent of a number of alkaloids. This metabolomic strategy assisted in delineating species in their chemotaxonomic groups despite the observation of morphological similarity amongst the species.

The use of metabolomics as a chemotaxonomic tool to provide a chemical-based signal to phylogenetic DNA studies is viewed as being powerful and complimentary, more especially in cases where genomic analyses converge with metabolomic patterns (47). Therefore, a phylogenetic analysis of the plants studied here may be imperative in future. Ernst et al. (48) looked into a phylogeny-guided drug discovery approach in *Euphorbia* species for anti-inflammatory phytochemicals (14). The study identified potential species within the genus that may have novel pharmaceutically valuable phytochemistry of interest based on phylogenetic signals (14). Pellicer et al. (49), did a similar study using phylogenetic to identify anti-antimalarial *Artemisia* species (49). The study presented findings suggesting that the artemisinin (malaria phytochemical) biosynthetic pathway may be an ancestral trait and the biosynthetic machinery required for the production of artemisinin may be common in several *Artemisia* species (49). This may be the case in the *Sceletium* genus, however the biosynthetic pathway of the mesembrine alkaloids has not fully been described but has been elucidated to some extent (2). The predictive power of phylogenetics in *Sceletium* may reveal a shared phytochemistry in the genus.

The alkaloid distribution was found to be variable both at the inter and intra-species levels. The chemical diversity observed between species of *S. tortuosum* (Figure 3) represents intra-species chemical variability, this can also be observed in the large variation in concentration of alkaloids (Figure 4). Large chemical variation within populations is suspected to be due to the vegetative islands in which *Sceletium* species tend to grow in. The plants tend to grow in vegetative island pockets with plants raised no higher than 5–10 cm off the ground. They are usually growing beneath a larger more established shrub that is not *Sceletium*, which offers it shade in the UV-intense environments that it grows in. Plants on the outer edge of the vegetative island are exposed to full sunlight conditions whilst those that grow beneath the shrub tend to be in full or semi-shaded environments. This may be a considerable contributor to the variability in alkaloid profiles. Large chemical differences may be arising as a result of a niche environment adaptive role.

Aside from solely geographical influences, phenotypic plasticity exists in organisms as a functional response to environmental stresses at a physiological, biochemical or morphological level (50), enabling plants to adapt to dynamic environments (37). The role of phenotypic plasticity may be to adapt to dynamic environments (37). Adaptive changes to the environment may alter the morphology and phytochemical distribution in plants even within the same population, due to highly plastic individuals (51, 52). The influence of heritable epigenetic alterations resultant from a variety of environmental pressures and growth conditions can confer phenotypic plasticity in plants occurring at different biogeographical scales (53–56). In other species with a widespread latitudinal distribution, for example in *Pilocarpus pennatifolius*, extensive variability in chemical profiles is possibly explained by bioregional factors across populations (57, 58) and stress-related epigenetic alterations that possibly become heritable from one generation to another likely have an influence in terms of *Sceletium* populations.

The chemical markers that were useful in distinguishing species of *Sceletium* were 4-(3,4-dimethyoxyphenyl)-4-[2- acetylmethlamino)ethyl]cyclohexanone (*S. tortuosum*), Sceletium A4 (*S. strictum*) and 4'-O-Demethylmesembrenol (*S. emarcidum*). The presence of cryptic species in the genus may be a potential contributor to the intra-population level chemical diversity evident in *S. tortuosum* populations (Figure 3). Application of structural metabolomics is fast gaining momentum to better characterize evolutionary relationships for species with cryptic chemical traits as inherent interspecific variability associated with specialized metabolism that may facilitate the coexistence of species, whilst driving evolutionary diversification patterns at the community ecology and macroevolutionary scales. Cryptic speciation has been hypothesized for several different genera such as *Bursera, Inga*, and *Piper*, summarized in the review of Sedio (59). The ecological establishment of cryptic species is at present not well understood but has been hypothesized to be driven by several biotic and abiotic events, including herbivory as species radiation may be aligned with adaptive radiations within a particular plant lineage (49, 59). This is, however, a new hypothesis and an analysis of the phylogenetic relationships within this taxon and at the population is currently unavailable.

### Feature-Based Molecular Networking of Different Populations

The lack of clear separation in the global analysis was solved by performing a supervised analysis on a selection of samples with the most variable chemistry. Crude extracts such as those used in this study are challenging to work with as they contain many unknown compounds whose identities remain undefined even when compound spectral libraries are used to search for identities. Molecular networks become useful for those MS/MS fragment ion spectra that are similar with metabolites that are eluting at different retention times (60).

**Figure 6:**
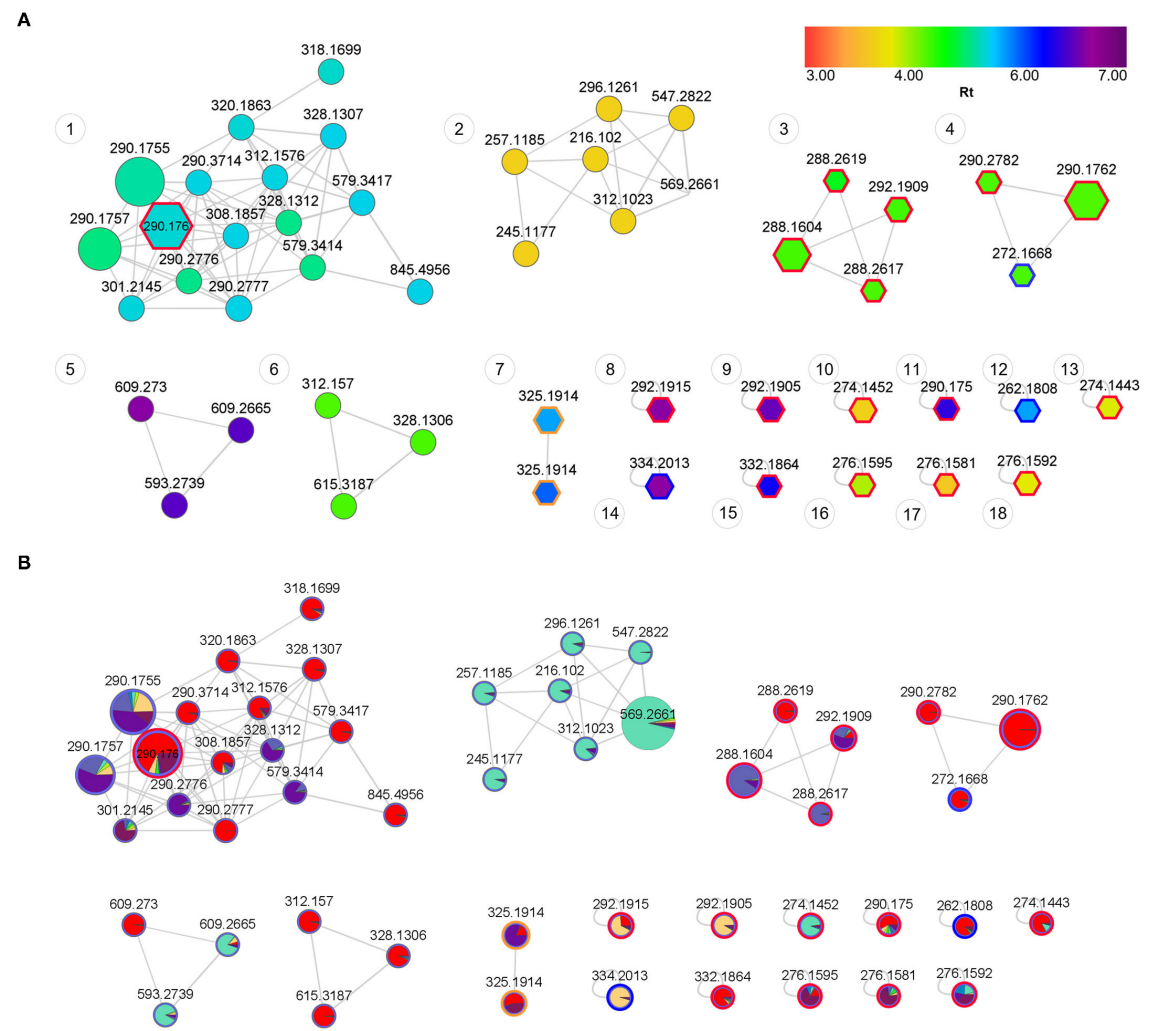
**(A)** Feature-based molecular network of *Sceletium* populations showing structural trends. Annotated nodes with pentagon boarders represent putatively identified metabolites (Table 2). Red outlines represent the mesembrine alkaloids, blue outlines represent joubertiamine alkaloids and orange outlines represent Sceletium A4 alkaloid class. Nodes are colored by retention time (RT) with their size representing the abundance of the metabolite. The distance between nodes indicates structural similarity based on fragmentation patterns from tandem mass spectrometry data. **(B)** Feature-based molecular network of *Sceletium* populations showing phytochemical distribution trends across populations. Red: *S. tortuosum* (De Rust); Blue: *S. strictum* (Anysberg); Green: *S. expansum* (Calitzdorp) and Purple: *S. rigidum* (Prince Albert).

Spectral analysis and manual inspection of MS data revealed that the clustering observed was according to alkaloid classes as determined by functional groups (Figure 6A). Molecular families were thus adequately separated, creating networks based on structural and fragmentation pattern (MS^2^) similarity between samples. Visualizing the chemical space with an overlay of retention time allows for the interpretation of chemical polarity (61). The largest cluster in the network (Figure 6A, Cluster 1) is largely made up of unknown alkaloids and the only alkaloid that could be putatively identified was mesembrine. There is still a paucity of information that would allow for a full chemical characterization of the alkaloid profile of *Sceletium*, and Cluster 1 is composed of new compounds whose chemical identities are thus presently unknown. With these novel metabolites grouping in close association to mesembrine, the network association implies that they are more structurally similar to mesembrine and may thus belong to this mesembrine class of alkaloids albeit occurring at possibly minute concentrations. Although molecular networking is a powerful dereplication tool for the annotation of “unknowns” resolved by MS ion fragmentation data, it is still challenged by the absence of fully annotated metabolite libraries as molecular networks are heavily reliant on existing MS/MS data in natural products repositories (62). The second network (Figure 6A, Cluster 2) had no compounds that could be tentatively identified. However, it was apparent that this molecular family of compounds was majorly distributed in *S. strictum*, supporting the separation of *S strictum* in the supervised PLS-DA (Figure 4). To our knowledge, there are no other investigations that have focused on *S. strictum* metabolomic trends. All studies have largely focused on *S. tortuosum* and recently the work of Patnala and Kanfer (63), also included an analysis of *S. emarcidum* where profiles were compared to *S. tortuosum* (63). Interestingly, the fourth molecular family (Figure 6A, Cluster 4) illustrated the association of mesembrenol isomers and O-methyldehydrojoubertiamine. The tentative identification of O-methyldehydrojoubertiamine was only made possible through the power of feature-based molecular networking that separated phytochemicals that were co-eluting with other metabolites, in particular those that are regarded as mesembrenol isomers. A chemical association that has not been suggested elsewhere and may hold some key information in understanding biosynthetic pathways within the genus that were last proposed on the work of Nieuwenhuis et al. (64). Using molecular network associations, we were thus able to infer tentative identifications of three joubertiamine type alkaloids, namely, O-methyldehydrojoubertiamine (*m/z* 272.1668), dihydrojoubertiamine (*m/z* 262.1808) and 4-(3,4-dimethyoxyphenyl)-4-[2-acetylmethlamino)ethyl]cyclohexanone (*m/z* 334.2014), and a set of isomeric Sceletium A4 alkaloids (Table 2). More importantly, no tortuosamine alkaloid class compounds were found in any of the populations (20, 63). The presence of tortuosamine alkaloids has been shown in previous studies and these are regarded as minor constituents of *Sceletium* species (65, 66), but their pharmacological function is still unknown.

The co-relation of locality and species data enabled powerful and robust downstream applications in that we could now identify unique chemical families and isomeric associations in different populations (Figure 6B). When comparing the four species of *Sceletium*, it is apparent that the most chemically diverse set of *S. tortuosum* plants were collected from De Rust. Interestingly, *S. tortuosum* and *S. strictum* share a great deal of metabolites but differ in the distribution of quantity of these alkaloids (indicated as red and blue clusters in Figure 6B). Morphologically, these species are different (Figures 1A,B, respectively) but occur within the same geographic distribution. However, these morphological differences are rather slight and may not be easily discernable leading to misidentification. Plants of *Sceletium* have been classified either as *emarcidum* or *tortuosum* morphological types based on venation patterns of dried leaves (63). *Emarcidum* types are flatter and show a clear central vein with curved secondary veins whereas the *tortuosum* morphotypes are distinguishable on the basis of dry leaves exhibiting a more concave central vein with 5–7 major parallel veins (63). Molecular networking in combination with metabolomic analyses may provide a tool to assist in distinguishing these species. Molecular networking revealed phytochemicals that may have been coeluting with other metabolites using the MS/MS spectra. These metabolites could then be selected and used for metabolite screening to identify populations that are potentially interesting from a pharmacological standpoint.

### Molecular Docking

Metabolomics and molecular networking enabled the identification of phytochemicals in *Sceletium* populations. Using these tools one can identify the suite of chemical diversity in a population. In a pharmacological context, *Sceletium* is used for mood elevation (1); understanding the alkaloid diversity in *Sceletium* is important as channeling the phytochemicals identified through molecular networking and metabolomics into an *in silico* analysis provided information on the predicted binding of compounds to biological targets modulating anxiety and depression. The combination of these experimental techniques can streamline the process of delivering anxiolytic and anti-depressive drugs.

**Figure 7:**
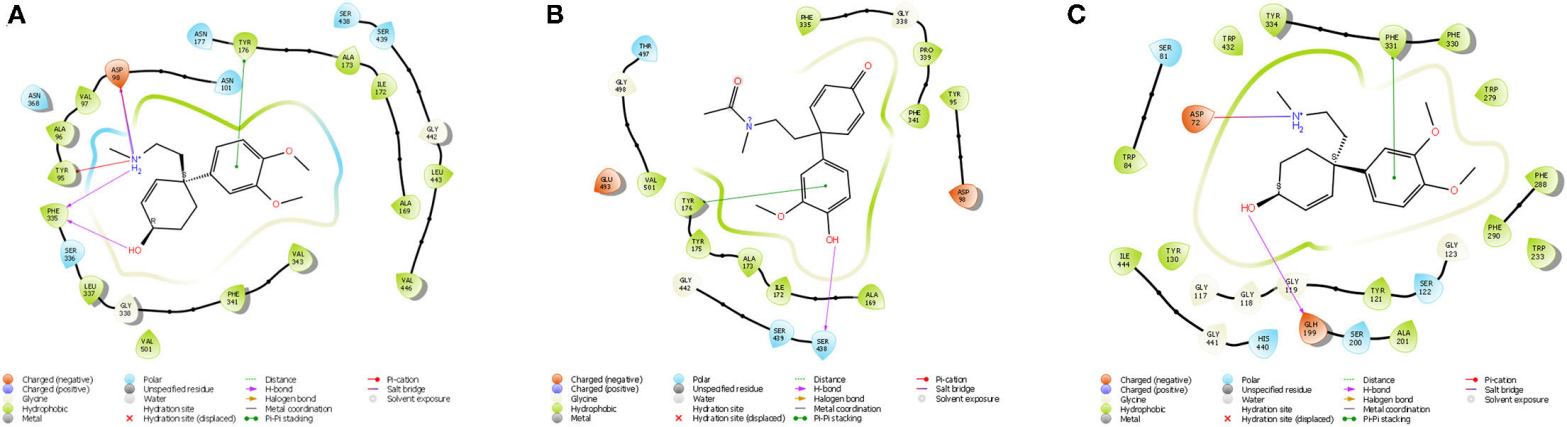
**(A)** Ligand interaction diagram of Dihydrojoubertiamine (-6.497 Δkcal/mol) in the flumazenil binding site on the GABA-A receptor; **(B)** Ligand interaction diagram of 4-(3-methoxy-4-hydroxy-phenyl)-4-[2-acetylmethylamino)ethyl]cyclohexadienone (-8.791Δkcal/mol) in the citalopram binding site on the 5-HT receptor; **(C)** Ligand interaction diagram of Mesembranol (-9.879Δkcal/mol) in the galantamine binding site on the AChE enzyme.

*In silico* molecular docking experiments were performed on three receptors responsible for the modulation of anxiety, depression, and cognitive enhancement (Tables 3–5). The conditions that were targeted in this study were selected in respect to the established pharmacological activities associated with specifically *S. tortuosum* as very little scientific *in vitro* or *in vivo* pharmacological information exists for the other species. Although ethnobotanical data indicates the use of *S. tortuosum*, these plants are difficult to differentiate in the wild from the other taxa of *Sceletium*. Often, plants that look same are not necessarily differentiated from each other at the ethnobotanical levels and are thus collected for use and may be regarded as a single ethnospecies (67). The mapping of ethnospecies and correlating these to scientific names is hence not always possible. For this reason, tentatively identified alkaloids from the *Sceletium* taxon sampled for this work were analyzed *in silico* and selected as molecular docking agents for binding to the serotonin receptor (5-HT) for depression and anxiety, the gamma aminobutyric acid (GABA) subtype A receptor for anxiety and fear, and the AChE enzyme for Alzheimer's. The best docked compounds were found to be 4-(3-methoxy-4-hydroxy-phenyl)-4-[2-acetylmethylamino)ethyl]cyclohexadienone (−8.791 Δkcal/mol), dihydrojoubertiamine (-6.497 Δkcal/mol) and mesembranol (−9.879 Δkcal/mol) against the 5-HT receptor, the GABA-A receptor, and the AChE enzyme, respectively, in static simulations (Figures 7A–C). This is interesting, as mesembrine that often occurs at high level in *S. tortuosum* (46, 68), has led to the assumption that this particular alkaloid may largely be responsible for pharmacological activity of this species. In this particular study, De Rust populations of *S. tortuosum* had considerably higher relative ion intensity values and these populations and/or species should thus be targeted for *in vitro* and *in vivo* pharmacological assays to validate the *in silico* data generated in the current study.

**Table 3:** Docking scores attained from docking XP scores (*in silico*) on serotonin transporter (GABA-A).

**Title**	**Binding free energy (Δkcal/mol)**
Flumazenil^*^	−7.016
Dihydrojoubertiamine	−6.497
Δ7-mesembrenone	−5.882
O-methyldehydrojoubertiamine	−5.756
4'-O-demethylmesembrenol	−5.727
Mesembranol	−5.214
Epimesembrenol	−5.208
Δ7-mesembrenone isomer	−5.033
(-)-Mesembrine	−4.722
O-methyldehydrojoubertiamine	−3.232

* *Positive control reference ligand*.

**Table 4:** Docking scores attained from docking XP scores (*in silico*) on serotonin transporter (5-HT).

**Title**	**Binding free energy (Δkcal/mol)**
Citalopram^*^	−9.787
4-(3-methoxy-4-hydroxy-phenyl)-4-[2-acetylmethylamino)ethyl]cyclohexadienone	−8.791
O-methyldehydrojoubertiamine	−7.842
Epimesembrenol	−7.647
O-Acetylmesembrenol	−7.642
Dihydrojoubertiamine	−7.57
Epimesembrenol	−7.068
Δ7-mesembrenone	−6.665
Mesembranol	−6.658
Δ7-mesembrenone	−6.152
4'-O-demethylmesembrenol	−6.116
O-methyldehydrojoubertiamine	−5.32
(-)-Mesembrine	−3.743

* *Positive control ligand*.

**Table 5:** Docking scores attained from docking XP scores (*in silico*) on Acetylcholinesterase enzyme.

**Title**	**Binding free energy (Δkcal/mol)**
Galantamine^*^	−10.423
Mesembranol	−9.879
Epimesembrenol	−9.685
Dihydrojoubertiamine	−9.089
O-methyldehydrojoubertiamine	−9.066
(-)-Mesembrine	−8.814
Epimesembranol	−8.448
Δ7-mesembrenone	−8.348
O-Acetylmesembrenol	−8.076
(+)-Mesembrine	−7.82
(+)-Mesembrenone	−7.352
*Sceletium* alkaloid A4	−6.27
(+)-Mesembrenone isomer	−6.054
4'-O-demethylmesembrenol	−4.64
*Sceletium* alkaloid A4 isomer	−4.436
Mesembrenol	−3.522

* *Positive control ligand*.

The docked compounds with GABA-A predominantly formed hydrogen bonds with the amino acid residues ASP-98 and PHE-335 in the binding pocket. The amino acid residue TYR-176 also created pi stacking (π-π stacking) with the benzene ring on ligands. These hydrogen bonds predominantly came from amine and hydroxyl groups on the phytochemicals. The most predominant amino acids in the binding site were, ASN-101, ASP-98, TYR-95, PHE-335 and VAL-343. The two-dimensional ligand interaction diagram for the best docked compound dihydrojoubertiamine (-6.497 Δkcal/mol, Table 3) is illustrated in Figure 7A. There have been no assays testing any alkaloids other than the mesembrine-type phytochemicals. Harvey et al. (22) reported little to no inhibition by isolated alkaloids (mesembrenol, mesembrenone and mesembrine alkaloids) on the GABA-A system. However, it was reported that the standardized extract Zembrin®, showed >80% inhibition of binding in that particular study. This may suggest that other phytochemicals in *Sceletium* may be responsible for the activity at the GABA-A receptor.

The docked compounds with 5-HT predominantly formed hydrogen bonds with the amino acid residue SER-438 in the binding pocket. Positive binding was observed with a number of alkaloids found in *Sceletium*, these were a combination of mesembrine and joubertiamine alkaloids (Table 4). These hydrogen bonds predominantly came from hydroxyl groups on the phytochemicals. The amino acid residue TYR-176 created π-π stacking with the benzene ring on ligands. The most predominant amino acids in the binding site were, GLY-338, PHE-341, SER-438, ALA-173 and TYR-176. The two-dimensional ligand interaction diagram for the best docked compound 4-(3-methoxy-4-hydroxy-phenyl)-4-[2-acetylmethylamino)ethyl]cyclohexadienone *(-*8.791Δ*kcal/mol*) is illustrated in Figure 7B. Harvey et al. (22) tested a standardized extract Zembrin® as well as mesembrenol, mesembrenone and mesembrine alkaloids against the 5-HT receptor and reported potent binding (IC_50_ 4.3 μg/ml) in the extract. In that particular study, all three alkaloids showed binding to the 5-HT system with Ki values of 1.4 nM, 27 nM and 62 nM for mesembrenol, mesembrenone and mesembrine, respectively. The results obtained by Harvey et al. (22) corroborate the predicted binding from *in silico* analyses in the current study (Table 4).

The docked compounds with acetylcholinesterase (AChE) predominantly formed hydrogen bonds with the amino acid residue ASP-72 and GLH-199 in the binding pocket. The amino acid residue PHE-331 also created π-π stacking with the benzene ring on ligands. Table 5 illustrates the trend that the most energetically favorable phytochemicals binding to this target belong to the mesembrine alkaloid class. These hydrogen bonds predominantly came from amine and hydroxyl groups on the phytochemicals. The most predominant amino acids in the binding site were, PHE-331, ASP-72, TYR-130, GLH-199, PHE-288 and PHE-290. The two-dimensional ligand interaction diagram for the best docked compound mesembranol (-9.879Δ*kcal/mol)* is illustrated in Figure 7C. The activity of *S. tortuosum* on AChE enzyme has been reported in several studies (22, 26, 69), however isolated alkaloids aside from mesembrine have not been tested in these assays. Harvey et al. (22) examined a *S. tortuosum* extract as well as isolated alkaloids of mesembrine, mesembrenone and mesembrenol against the AChE system. The results showed none of the isolated alkaloids reduced activity by >5–10% and extracts tested at 300 μg/ml reduced AChE activity by 7%. The work of Lubbe et al. (26) assessed a fermented and unfermented methanol extract of *S. tortuosum* on the AChE enzyme system and found IC_50_ values of 0.303 mg/ml and 0.330 mg/ml, respectively. The pure mesembrine extract did not show inhibition, suggesting that this major alkaloid may not be involved in this biological system (26). A study conducted by Bennet et al. (69) analyzed the inhibitory activity of a mesembrine-rich extract against a Δ7-mesembrenone-rich extract and observed that the mesembrine-rich extract displayed a higher degree of potency than the Δ7-mesembrenone-rich extract (69). Of the *in vitro* assays performed on AChE, no tests have been conducted on mesembranol which showed the highest binding free energy in docking studies (-9.879 Δkcal/mol). Another plausible explanation of poor activity observed in isolated extracts may allude to the possibility of multiple phytochemicals playing a role in eliciting neurological responses related to neurological dysfunctions such as Alzheimer's (70).

A fascinating observation in all three docking experiments was that joubertiamine alkaloids showed binding scores comparable to positive controls (Tables 3–5). This is the first evidence put forward that minor alkaloids may be responsible for neurological activity observed *in vitro*. Current reports where isolated extracts have negligible activity as compared to extracts may suggest other phytochemicals responsible for the mood-elevation and cognitive enhancement activity in *Sceletium* (1, 22). Further *in vitro* and *in vivo* studies would have to be conducted on isolated metabolites to corroborate these findings.

This part of the study is thus proposing the application of other *Sceletium* species which have higher amounts of the metabolites that bind significantly to the test receptors. This may add valuable insights to the function of the other metabolites apart from mesembrine in the biological effects of *Sceletium* plants. Such data may be important, as the study of Coetzee et al. (71) which concluded that plants with a lower fraction of mesembrine-type alkaloids should be assayed in biological studies as the high mesembrine-fractions, function more efficiently through monoamine oxidase-A (MAO-A) inhibition activity rather than inhibiting serotonin-reuptake. Results from this study indicate that not only do different species also contain alkaloids of interest but phytochemical profiles differ geographically. The *in silico* analyses in this study have indicated that multiple receptors with observed *in vitro* activity (22) have several phytochemicals from *Sceletium* that may effectively bind to them. The introduction of *Sceletium* as a daily nutritive supplement may hold potential in promoting health and treating a variety of neurological pathologies. Currently there is very little information on the development of other species in the *Sceletium* genus as a nutritive supplement. Results from the current present the potential of *S. strictum* as a nutritive supplement to aid with anxiety and depression.

## Conclusions

To test the hypothesis that different *Sceletium* species would exhibit metabolites that could be used as distinguishing features, a chemotaxonomic perspective was explored in this study. Gaining an understanding of how metabolites differ both between and within species provides valuable information in relation to the potential utilization of these species to manufacture dietary supplements for the complementary and alternative medicines sector. Many of the species were qualitatively similar but often differed in quantities of both major and minor alkaloids and sometimes, some key chemical markers were absent in species such as *S. rigidum*. Intra-population metabolomic analyses between *S. tortuosum* revealed some populations that were chemically distinct. As a novel approach developed for *Sceletium* and its mesembrine alkaloids, the use of feature-based molecular enabled a greater degree of separation and dereplication of mesembrine alkaloids and for the first time, we show the occurrence of O-methyldehydrojoubertiamine that co-eluted with mesembrenol in *S. tortuosum* found in De Rust. This result is indicative of the resolving power of molecular network associations, more especially for metabolites that remain masked in a chromatographic landscape that have similar elution retention times and mass ion fragmentation patterns. It would be important in the future to test their pharmacological activities using *in vitro* and *in vivo* bioassays that target those metabolites that have been shown here to have strong *in silico* receptor-binding affinities. Performing these assays on selected metabolites may aid in verifying whether these specific phytochemicals can assist with neurological disorders such as anxiety and depression. To this end, Kannaland (*S. tortuosum*), De Rust (*S. tortuosum*) and Anysberg (*S. strictum*) should be included in those studies due to their inherent capacity to accumulate higher amounts of mesembranol (Kannaland), dihydrojoubertiamine (De Rust), and 4-(3-methoxy-4-hydroxy-phenyl)-4-[2-acetylmethylamino)ethyl]cyclohexadienone (De Rust). The Anysberg chemotype with broad alkaloid diversity might be of higher value for the phytopharmaceutical industries. These metabolites were amongst the most promising molecular docking targets for GABA-A, AChE and 5-HT respectively. The joubertiamine and Sceletium A4 alkaloid classes hold potential in aiding in anxiety and depression. Further investigation into the pharmacological activity of other *Sceletium* species aside from *S. tortuosum* is needed. In the future, molecular-based phylogenetic resolution may also provide fundamental information regarding chemical lineages of *Sceletium* indicated in the present work.

## Data Availability Statement

The original contributions presented in the study are included in the article/Supplementary Materials, and MS data were deposited in the Global Natural Products Social Molecular Networking (GNPS) as a MassIVE dataset (accession number: MSV000088410).

## Author Contributions

KR performed all experiments, conducted the data analyses, and compilation. KR and NM wrote the first draft of this manuscript. MAS performed the LC-MS analyses and validation, assisted with interpretation of MSE spectra, and edited the first draft. GS and NM conceptualized the study and contributed by editing the draft versions of this paper. All authors read and approved the final version of this review article.

## Funding

This study was financed by the National Research Foundation of South Africa (grant number: 76555) awarded to NM. KR is a recipient of the NRF-Foundational Biodiversity Information Programme (FBIP) for 2020-2021. GS is a recipient of a Medical Research Council (South Africa) Self-initiated Research (SIR) grant entitled validating the anticonvulsant action of African plant extracts. The funders are thus thanked for their financial contributions.

## Conflict of Interest

The authors declare that the research was conducted in the absence of any commercial or financial relationships that could be construed as a potential conflict of interest.

## Publisher's Note

All claims expressed in this article are solely those of the authors and do not necessarily represent those of their affiliated organizations, or those of the publisher, the editors and the reviewers. Any product that may be evaluated in this article, or claim that may be made by its manufacturer, is not guaranteed or endorsed by the publisher.
