# Corrigendum: Mass spectrometry metabolomics and feature-based molecular networking reveals population-specific chemistry in some species of the *Sceletium* genus

**DOI:** 10.3389/fnut.2022.994203

**Published:** 2022-09-08

**Authors:** Kaylan Reddy, Marietjie A. Stander, Gary I. Stafford, Nokwanda P. Makunga

**Affiliations:** ^1^Department of Botany and Zoology, Faculty of Natural Sciences, Stellenbosch University, Stellenbosch, South Africa; ^2^Department of Biochemistry, Faculty of Natural Sciences, Stellenbosch University, Stellenbosch, South Africa; ^3^Department of Plant and Soil Sciences, University of Pretoria, Pretoria, South Africa

**Keywords:** alkaloid chemistry, eco-metabolomics, kanna, kougoed, mesembrine, molecular networks

In the published article, there was an error. The data referred to needs to correspond to Figure 5. A correction has been made to **Results and Discussion**, “*Chemical Profiling of Sceletium Populations*,” Paragraph number 1.

**Figure 5:**
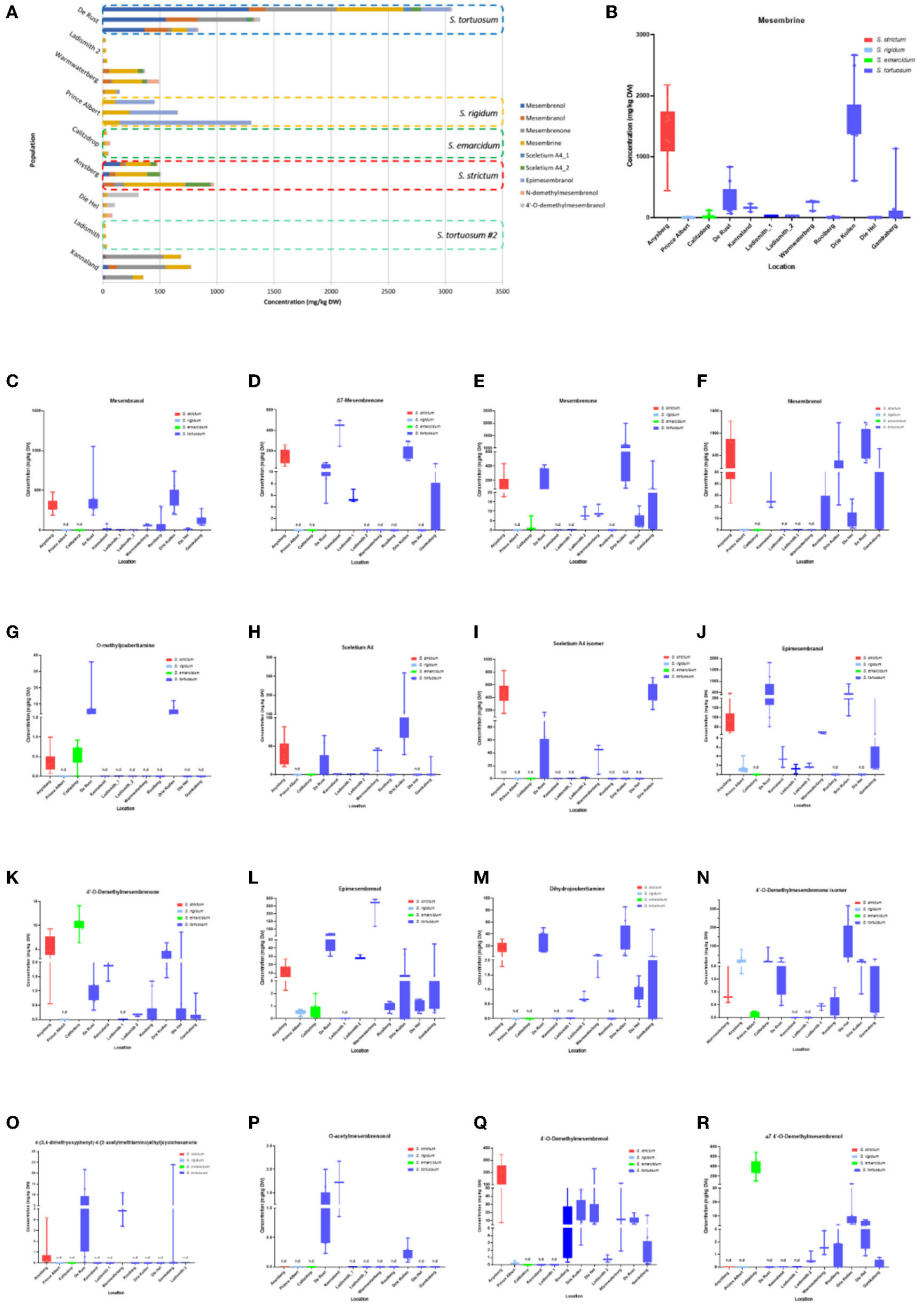
**(A)** Concentration of *Sceletium* metabolites in different species and populations found at twelve locations. Horizontal stacked columns represent metabolites (mg/kg DW) tentatively identified chemical markers responsible for metabolomic separations. Each horizontal stacked column represents up to 10 individuals per location and/or taxon *S. tortuosum* B-R) Concentration of tentatively identified metabolites (mg/kg DW) in *S. strictum, S. emarcidum, S. tortuosum* and *S. emarcidum* found at different locations: **(B)** Mesembrine; **(C)** Mesembranol; **(D)** Δ7-Mesembrenone; **(E)** Mesembrenone; **(F)** Mesembrenol; **(G)** O-methyldehydrojoubertiamine; **(H)** Sceletium A4; **(I)** Sceletium A4 isomer; **(J)** Epimesembranol; **(K)** 4'-O-Demethylmesembrenone; **(L)** Epimesembrenol; **(M)** Dihydrojoubertiamine; **(N)** 4'-O-Demethylmesembrenone isomer; **(O)** 4-(3,4-dimethyoxyphenyl)-4-[2-acetylmethlamino)ethyl]cyclohexanone; **(P)** O-acetylmesembrenol; **(Q)** 4'-O-demethylmesembrenol; **(R)** Δ7 4'-O-Demethylmesembrenol.

This sentence previously stated:

“These metabolites, specifically dihydrojoubertiamine, 4-(3,4-dimethyoxyphenyl) 4-[2-acetylmethlamino)ethyl]cyclohexanone, mesembrenol and 4'-O-demethylmesembrenol, were highest in Drie Kuilen (209674± 156580 mg/kg DW) (Figure 5M), Warmwaterberg (59,805 ± 37,239 mg/kg DW; *p* < 0.0001) (Figure 5O), Kannaland (319,373 ± 198,464 mg/kg DW) (Figure 5F) and Warmwaterberg (388,263 ± 565,782 mg/kg DW) (Figure 5Q), respectively.”

The corrected sentence appears below:

“These metabolites, specifically dihydrojoubertiamine, 4-(3,4-dimethyoxyphenyl) 4-[2-acetylmethlamino)ethyl]cyclohexanone, mesembrenol and 4'-O-demethylmesembrenol, were highest in Drie Kuilen (34.63 ± 25.86 mg/kg DW) (Figure 5M), Warmwaterberg (6.359 ± 3.964 mg/kg DW; *p* < 0.0001) (Figure 5O), De Rust (2.795 ± 0.2072 mg/kg DW) (Figure 5F) and Anysberg (181.2 ± 105.4 mg/kg DW) (Figure 5Q), respectively.”

In the published article, there was an error. The data referred to needs to correspond to Figure 5. A correction has been made to **Results and Discussion**, “*Chemical Profiling of Sceletium Populations*,” Paragraph number 2.

This sentence previously stated:

“A bi-plot of the PCA (Figure 3C) indicated that the major contributors to separation of the Kannaland population was mesembrenol (319,373 ± 198,464 mg/kg DW; Figure 5F) and Δ7-mesembrenone (3,737,464 ± 1,257,877 mg/kg DW; Figure 5D) and these were statistically significant (*p* < 0.0001).”

The corrected sentence appears below:

“A bi-plot of the PCA (Figure 3C) indicated that the major contributors to separation of the Kannaland population was mesembrenol (1.480 ± 0.2515 mg/kg DW; Figure 5F) and Δ7-mesembrenone (397.8 ± 133.9 mg/kg DW; Figure 5D) and these were statistically significant (*p* < 0.0001).”

In the published article, there was an error. The data referred to needs to correspond to Figure 5. A correction has been made to **Results and Discussion**, “*Chemical Profiling of Sceletium Populations*,” Paragraph number 6.

This sentence previously stated:

“Eighteen different alkaloids were tentatively identified using MSE fragmentation patterns, relative retentions times and accurate mass spectra and several of these metabolites were quantitatively higher in some of the populations, namely, Kannaland (*S. tortuosum*) and Ladismith 1 exhibiting higher amounts of Δ7-mesembrenone (m/z 288.1600) concentrations of 3,737,464 ± 1,257,877 mg/kg DW (*p* < 0.0001). Mesembrine (m/z of 290.1757), that is used as a chemical marker in manufactured products of *S. tortuosum* (1, 10), was highest in the plants collected from Warmwaterberg (2,020,055 ± 864,952/kg DW) and Kannaland (1,541,227 ± 614,992/kg DW). Sceletium A4 (m/z of 325.1914) that is structurally different from mesembrine by having a 2,3-disubstituted pyridine moiety and 2 nitrogen atoms, occurred in highest relative ion intensity in those plants that were collected from Warmwaterberg (324,398 ± 227,304/kg DW; *p* < 0.0001).”

The corrected sentence appears below:

“Eighteen different alkaloids were tentatively identified using MS^E^ fragmentation patterns, relative retentions times and accurate mass spectra and several of these metabolites were quantitatively higher in some of the populations, namely, Kannaland (*S. tortuosum*) and Ladismith 1 exhibiting higher amounts of Δ7-mesembrenone (m/z 288.1600) concentrations of 397.8 ± 133.9 mg/kg DW (*p* < 0.0001). Mesembrine (m/z of 290.1757), that is used as a chemical marker in manufactured products of *S. tortuosum* (1, 10), was highest in the plants collected from Drie Kuilen (1,640 ± 582.3 mg/kg DW) and Anysberg (1,402 ± 504.8 mg/kg DW). Sceletium A4 (m/z of 325.1914) that is structurally different from mesembrine by having a 2,3- disubstituted pyridine moiety and 2 nitrogen atoms, occurred in highest relative ion intensity in those plants that were collected from Drie Kuilen (114.5 ± 63.98 mg/kg DW; *p* < 0.0001).”

In the published article, there was an error. The data referred to needs to correspond to Figure 5. A correction has been made to **Results and Discussion**, “*Chemical Profiling of Sceletium Populations*,” Paragraph number 7.

This sentence previously stated:

“Joubertiamine alkaloids had a higher distribution in *S. tortuosum* species collected from Warmwaterberg and De Rust. The joubertiamine alkaloid 4-(3,4-dimethyoxyphenyl) 4-[2-acetylmethlamino)ethyl]cyclohexanone was found in concentrations of 59,805 ± 37,239 mg/kg DW and 257.3± 292.0 mg/kg DW (Figure 5O), respectively in these populations. *S. rigidum* (Prince Albert) had considerably lower levels of alkaloids than the other species. In the study of Patnala and Kanfer (46) samples of *S. rigidum* were reported to not have any mesembrine alkaloids. This particular species is morphologically different from all the other species in the genus (Figures 1D,E) as it has an upright form with many prominent idioblasts and a highly restricted distribution. In this study, it could easily be distinguished from the other *Sceletium* collections due to the absent of a number of alkaloids. This metabolomic strategy assisted in delineating species in their chemotaxonomic groups despite the observation of morphological similarity amongst the species.”

The corrected sentence appears below:

“Joubertiamine alkaloids had a higher distribution in *S. tortuosum* species collected from Warmwaterberg and De Rust. The joubertiamine alkaloid 4-(3,4-dimethyoxyphenyl) 4-[2-acetylmethlamino)ethyl]cyclohexanone was found in concentrations of 6.359 ± 3.964 mg/kg DW and 5.533 ± 6.848 mg/kg DW (Figure 5O), respectively in these populations. *S. rigidum* (Prince Albert) had considerably lower levels of alkaloids than the other species. In the study of Patnala and Kanfer (46) samples of *S. rigidum* were reported to not have any mesembrine alkaloids. This particular species is morphologically different from all the other species in the genus (Figures 1D,E) as it has an upright form with many prominent idioblasts and a highly restricted distribution. In this study, it could easily be distinguished from the other *Sceletium* collections due to the absent of a number of alkaloids. This metabolomic strategy assisted in delineating species in their chemotaxonomic groups despite the observation of morphological similarity amongst the species.”

In the original article, there was a mistake in Figure 5 as published. An older version of Figure 5 ended up uploaded. The corrected Figure 5 appears below.

The authors apologize for these errors and state that this does not change the scientific conclusions of the article in any way. The original article has been updated.

## Publisher's note

All claims expressed in this article are solely those of the authors and do not necessarily represent those of their affiliated organizations, or those of the publisher, the editors and the reviewers. Any product that may be evaluated in this article, or claim that may be made by its manufacturer, is not guaranteed or endorsed by the publisher.

