# Supplementary material for: Mass Spectrometry Metabolomics and Feature-Based Molecular Networking Reveals Population-Specific Chemistry in Some Species of the Sceletium Genus
Source: Front Nutr. 2022 Mar 29;9:819753. doi: 10.3389/fnut.2022.819753 (PMC9001948; doi:10.3389/fnut.2022.819753)
Supplement: Supplementary file 1 [file Data_Sheet_1.PDF]

Supplementary A

**Chemical Structures**

(-)- Mesembrine

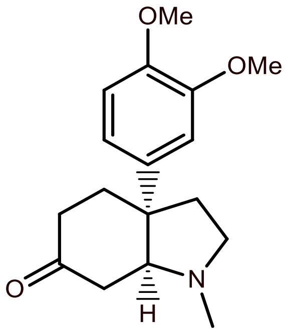


Chemical Formula: $C_{17}H_{23}NO_3$
Exact Mass: 289.1678
Molecular Weight: 289.3694
m/z: 289.1678 (100.0%), 290.1711 (18.4%), 291.1745 (1.6%)
Elemental Analysis: C, 70.56; H, 8.01; N, 4.84; O, 16.59

Mesembrenone

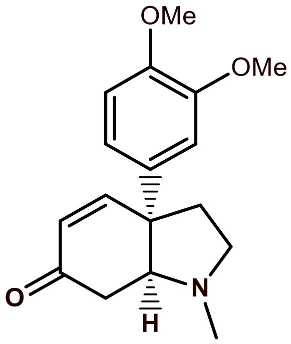


Chemical Formula: $C_{17}H_{21}NO_3$
Exact Mass: 287.1521
Molecular Weight: 287.3535
m/z: 287.1521 (100.0%), 288.1555 (18.4%), 289.1589 (1.6%)
Elemental Analysis: C, 71.06; H, 7.37; N, 4.87; O, 16.70

D7-Mesembrenone

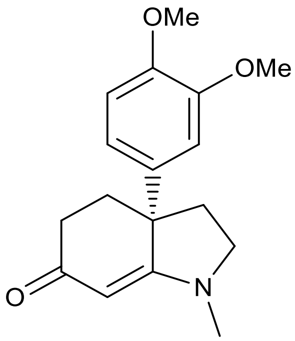


Chemical Formula: $C_{17}H_{21}NO_3$
Exact Mass: 287.1521
Molecular Weight: 287.3535
m/z: 287.1521 (100.0%), 288.1555 (18.4%), 289.1589 (1.6%)
Elemental Analysis: C, 71.06; H, 7.37; N, 4.87; O, 16.70

Mesembrenol

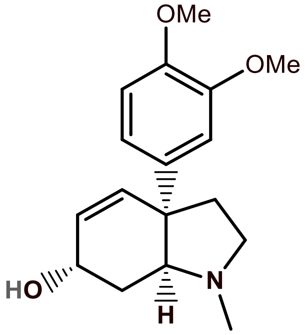


Chemical Formula: $C_{17}H_{23}NO_3$
Exact Mass: 289.1678
Molecular Weight: 289.3694
m/z: 289.1678 (100.0%), 290.1711 (18.4%), 291.1745 (1.6%)
Elemental Analysis: C, 70.56; H, 8.01; N, 4.84; O, 16.59

epimesembranol

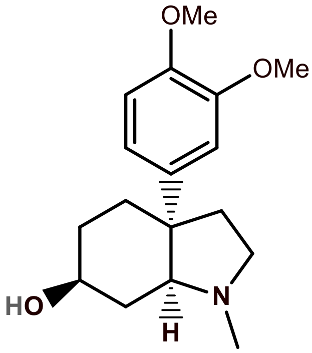


Chemical Formula: $C_{17}H_{25}NO_3$
Exact Mass: 291.1834
Molecular Weight: 291.3853
m/z: 291.1834 (100.0%), 292.1868 (18.4%), 293.1902 (1.6%)
Elemental Analysis: C, 70.07; H, 8.65; N, 4.81; O, 16.47

Epimesembrenol

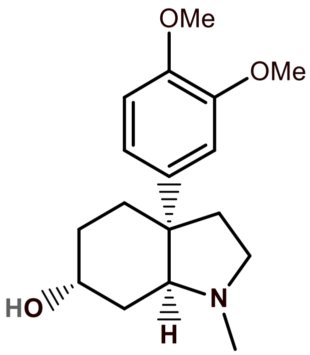


Chemical Formula: $C_{17}H_{25}NO_3$
Exact Mass: 291.1834
Molecular Weight: 291.3853
m/z: 291.1834 (100.0%), 292.1868 (18.4%), 293.1902 (1.6%)
Elemental Analysis: C, 70.07; H, 8.65; N, 4.81; O, 16.47

dihydrojoubertiamine

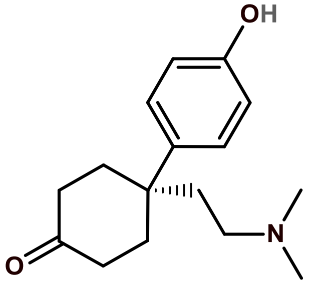


Chemical Formula: $C_{16}H_{23}NO_2$
Exact Mass: 261.1729
Molecular Weight: 261.3593
m/z: 261.1729 (100.0%), 262.1762 (17.3%), 263.1796 (1.4%)
Elemental Analysis: C, 73.53; H, 8.87; N, 5.36; O, 12.24

O-methyldehydrojoubertiamine

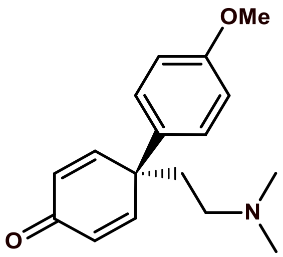


Chemical Formula: $C_{17}H_{21}NO_2$
Exact Mass: 271.1572
Molecular Weight: 271.3541
m/z: 271.1572 (100.0%), 272.1606 (18.4%), 273.1639 (1.6%)
Elemental Analysis: C, 75.25; H, 7.80; N, 5.16; O, 11.79

4'-O-methylmesembrenone

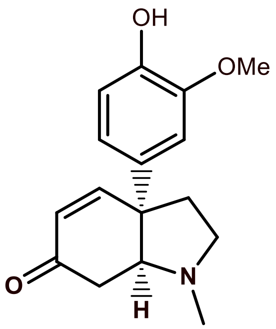


Chemical Formula: $C_{16}H_{19}NO_3$
Exact Mass: 273.1365
Molecular Weight: 273.3270
m/z: 273.1365 (100.0%), 274.1398 (17.3%), 275.1432 (1.4%)
Elemental Analysis: C, 70.31; H, 7.01; N, 5.12; O, 17.56

4'-O-demethylmesembranol

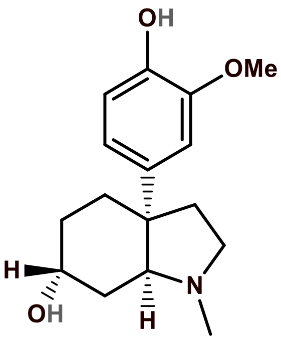


Chemical Formula: $C_{16}H_{23}NO_3$
Exact Mass: 277.1678
Molecular Weight: 277.3587
m/z: 277.1678 (100.0%), 278.1711 (17.3%), 279.1745 (1.4%)
Elemental Analysis: C, 69.29; H, 8.36; N, 5.05; O, 17.31

4'-O-demethylmesembrenol

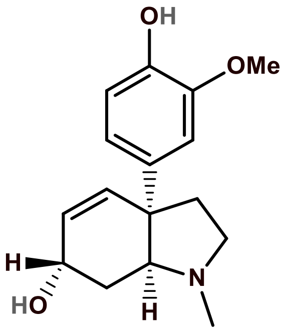


Chemical Formula: $C_{16}H_{21}NO_3$
Exact Mass: 275.1521
Molecular Weight: 275.3428
m/z: 275.1521 (100.0%), 276.1555 (17.3%), 277.1589 (1.4%)
Elemental Analysis: C, 69.79; H, 7.69; N, 5.09; O, 17.43

O-Acetylmesembrenol

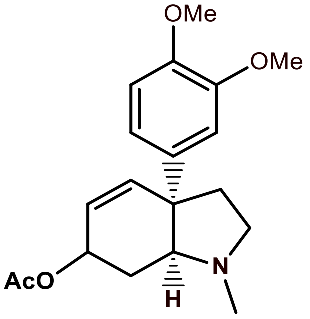


Chemical Formula: $C_{19}H_{25}NO_4$
Exact Mass: 331.1784
Molecular Weight: 331.4061
m/z: 331.1784 (100.0%), 332.1817 (20.5%), 333.1851 (2.0%)
Elemental Analysis: C, 68.86; H, 7.60; N, 4.23; O, 19.31

4-(3,4-dimethoxyphenyl)-4-[2-acetylmethylamino)ethyl]cyclohexane

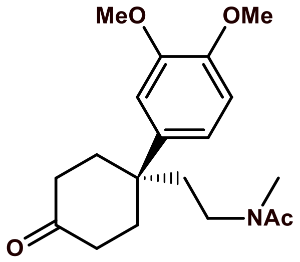


Chemical Formula: $C_{19}H_{27}NO_4$
Exact Mass: 333.1940
Molecular Weight: 333.4220
m/z: 333.1940 (100.0%), 334.1974 (20.5%), 335.2007 (2.0%)
Elemental Analysis: C, 68.44; H, 8.16; N, 4.20; O, 19.19

Sceletium alkaloid A4

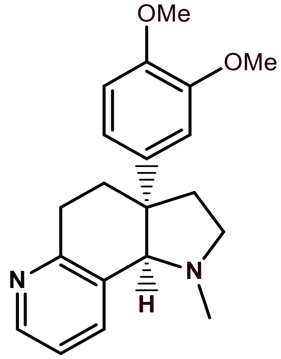


Chemical Formula: $C_{20}H_{24}N_2O_2$
Exact Mass: 324.1838
Molecular Weight: 324.4168
m/z: 324.1838 (100.0%), 325.1871 (21.6%), 326.1905 (2.2%)
Elemental Analysis: C, 74.04; H, 7.46; N, 8.64; O, 9.86
