# Supplementary material for: Mass Spectrometry Metabolomics and Feature-Based Molecular Networking Reveals Population-Specific Chemistry in Some Species of the Sceletium Genus
Source: Front Nutr. 2022 Mar 29;9:819753. doi: 10.3389/fnut.2022.819753 (PMC9001948; doi:10.3389/fnut.2022.819753)

Supplementary B

Full Chromatograms

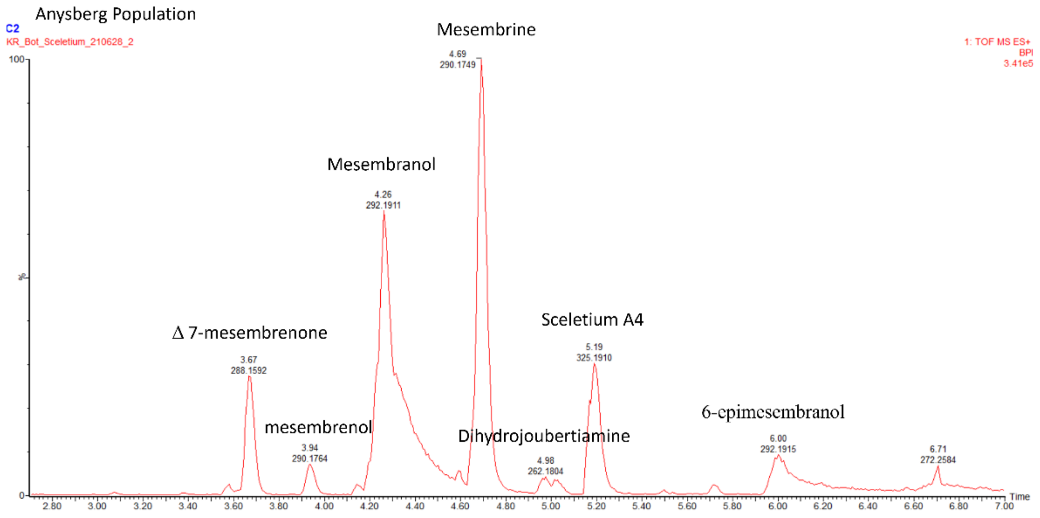

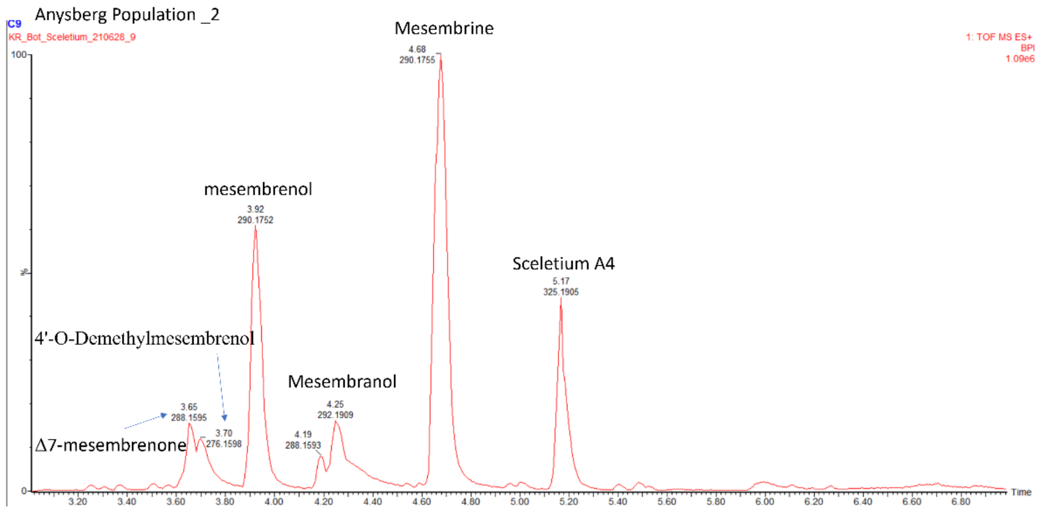

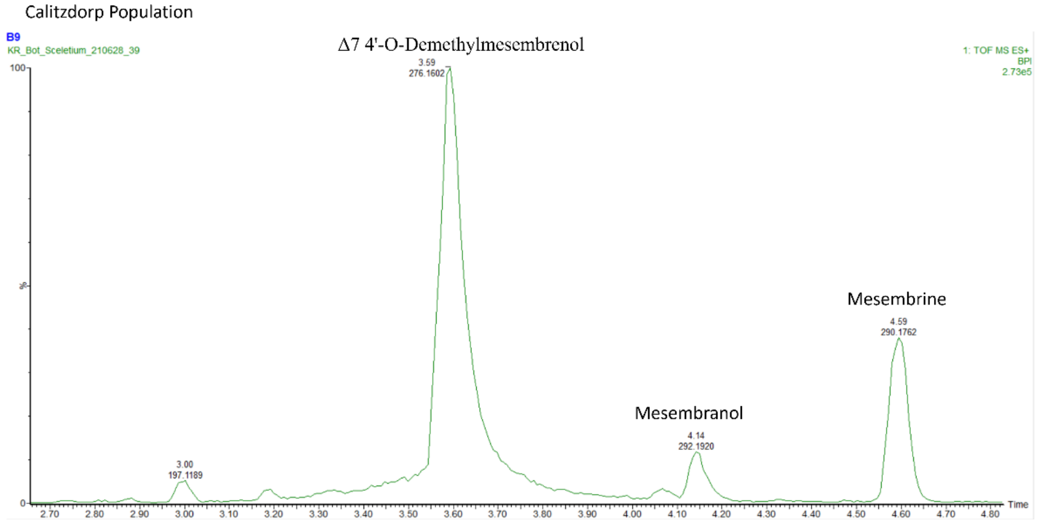

De Rust Population

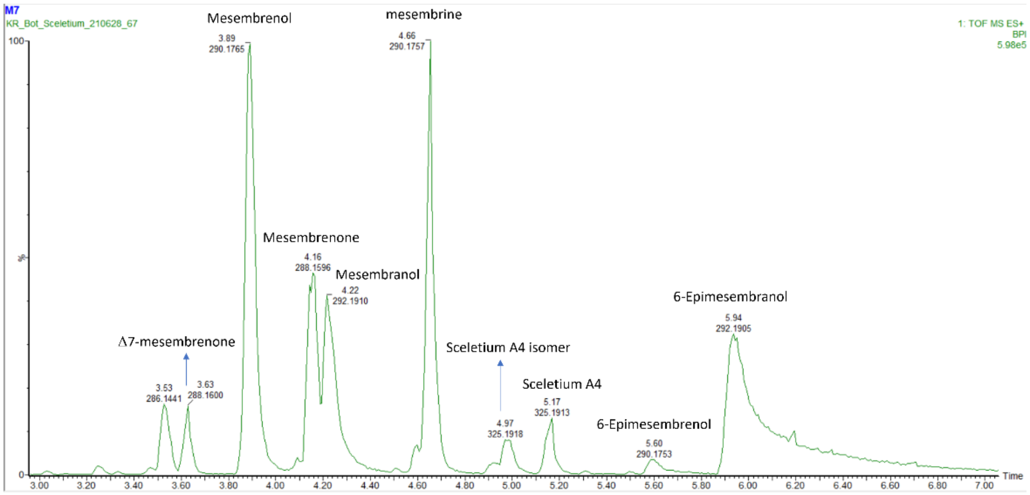

Caltzdorp Population

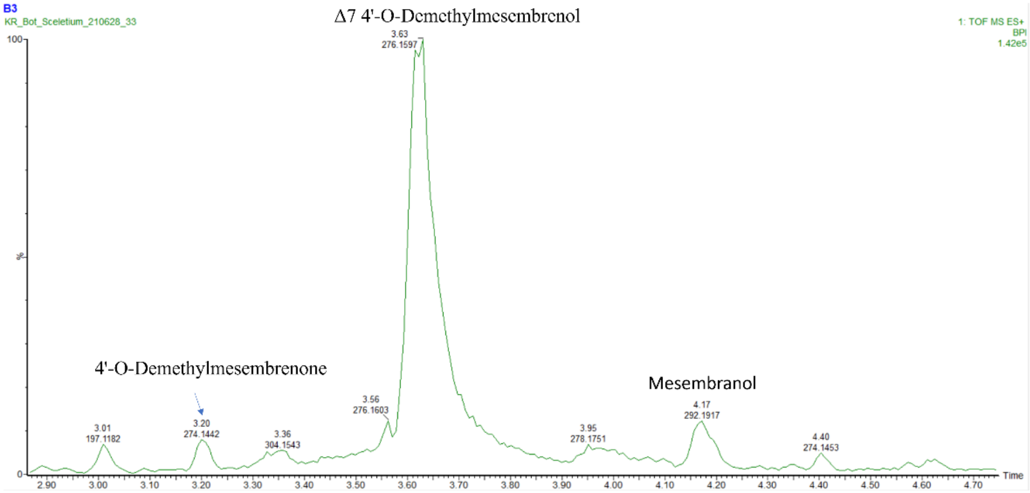

Die Hel Population

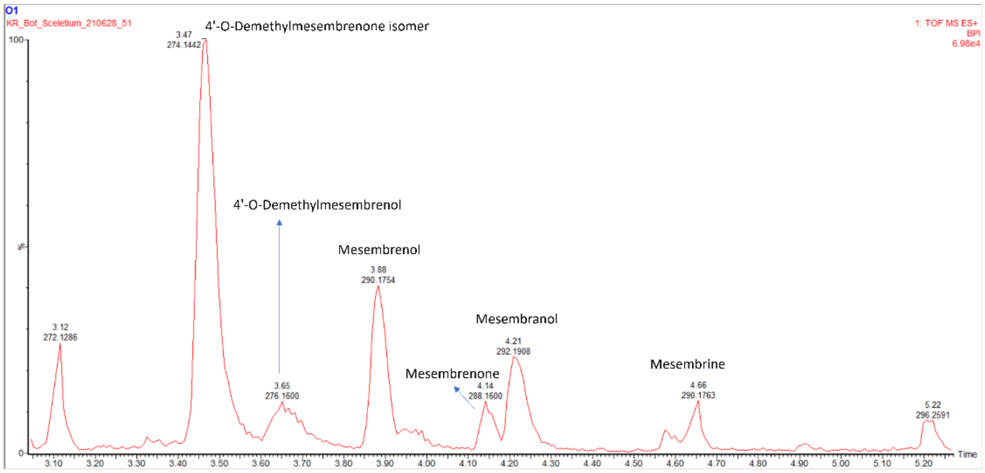

Anysberg population

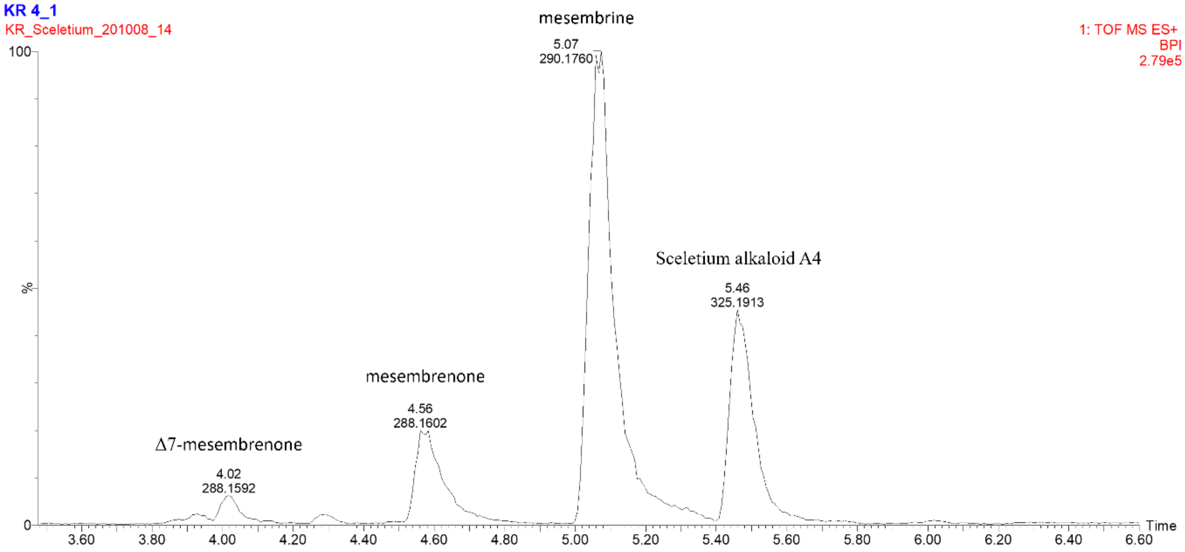

De Rust population

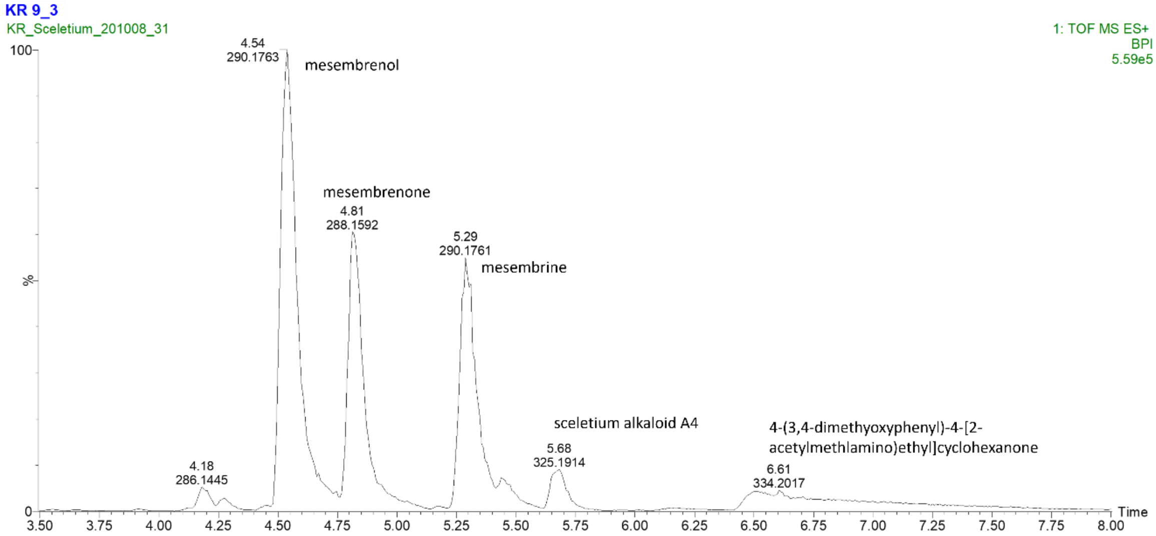

Anysberg population

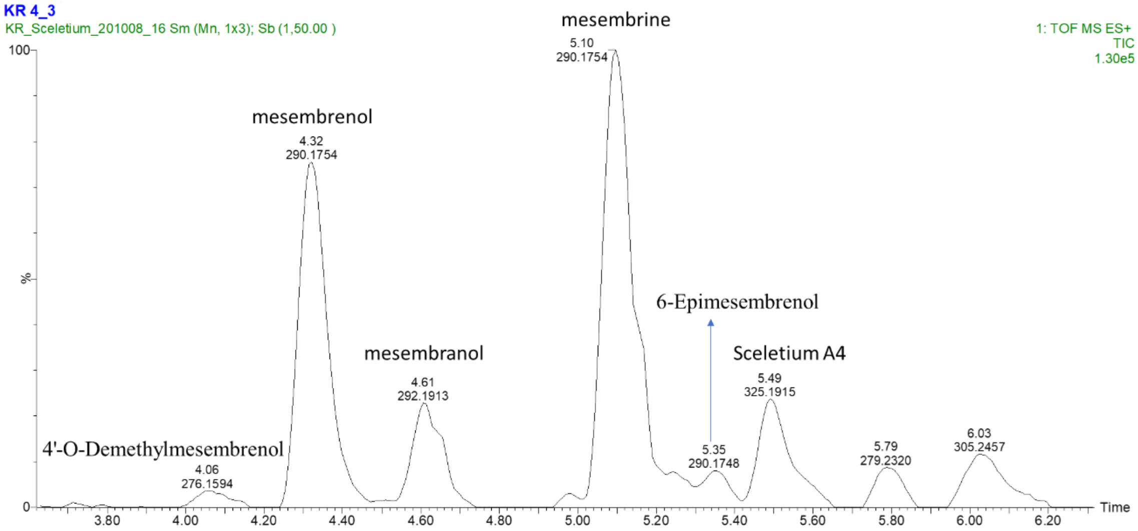

Kannaland population

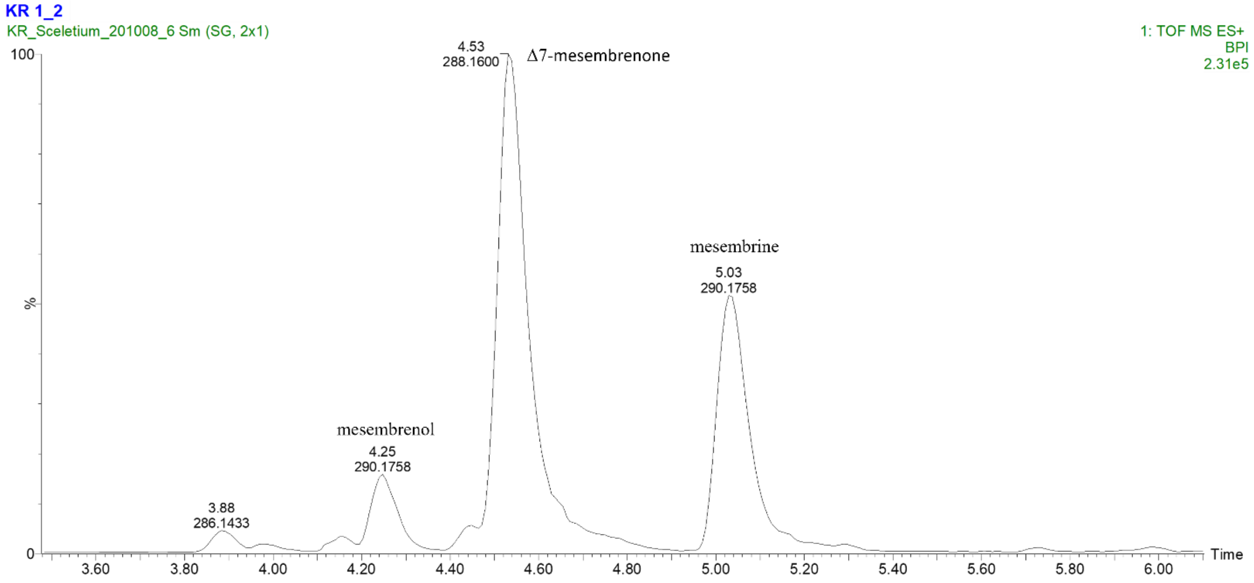

Warmwatreberg population

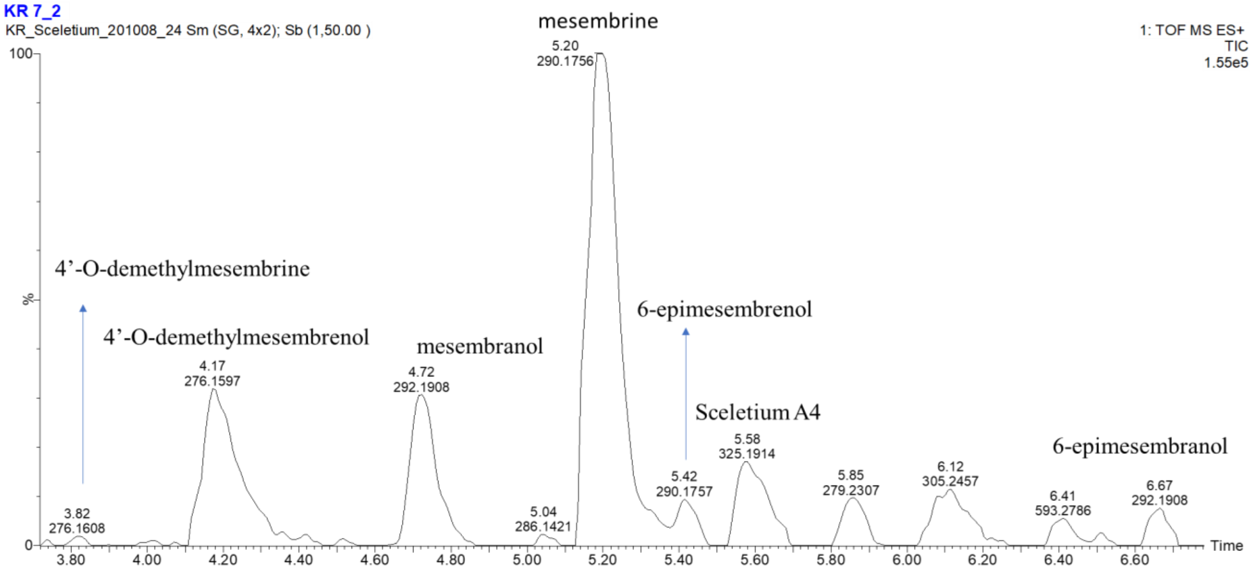

Calitzdorp population

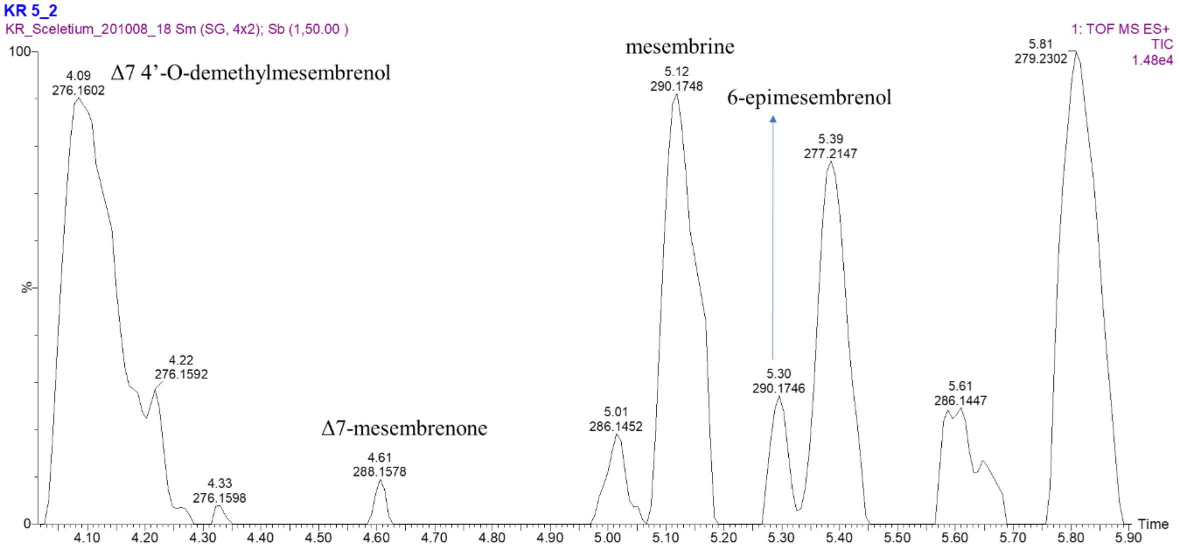

De Rust population

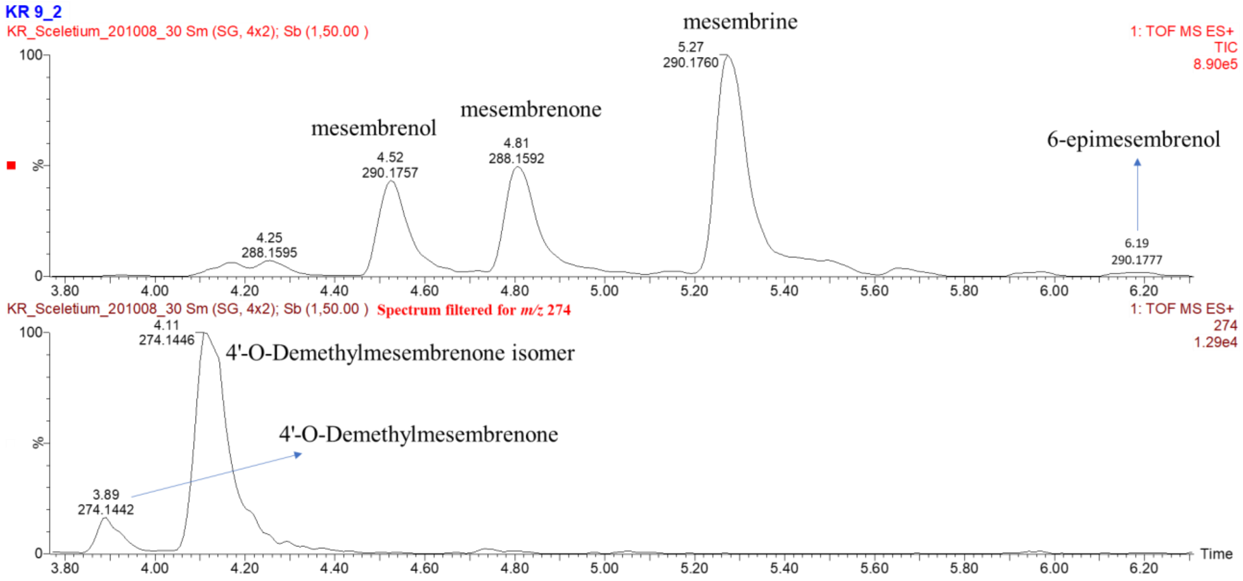

Fragmentation spectra

Spectral data for mesembrine

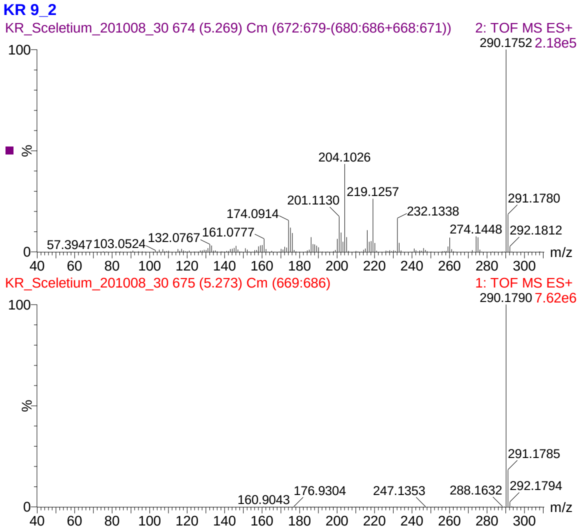

Spectral data for mesembrenone

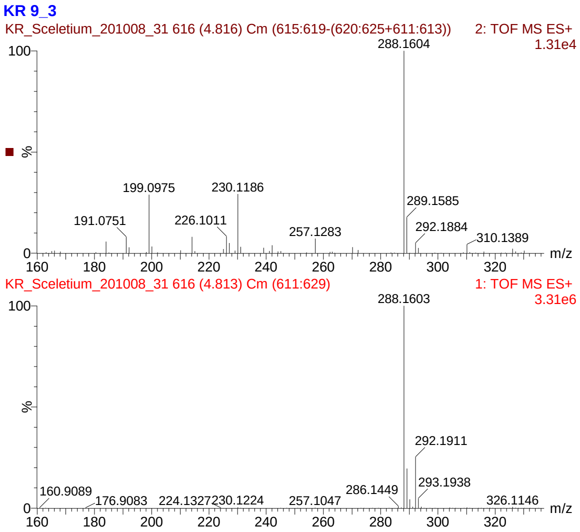

Spectral data for Δ7-mesembrenone

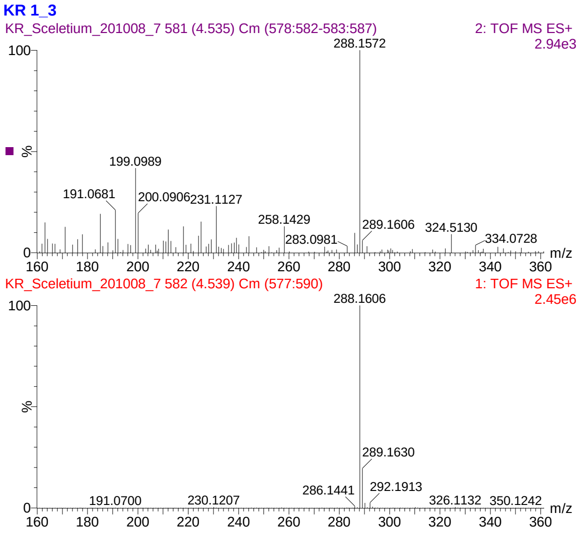

Spectral data for mesembrenol

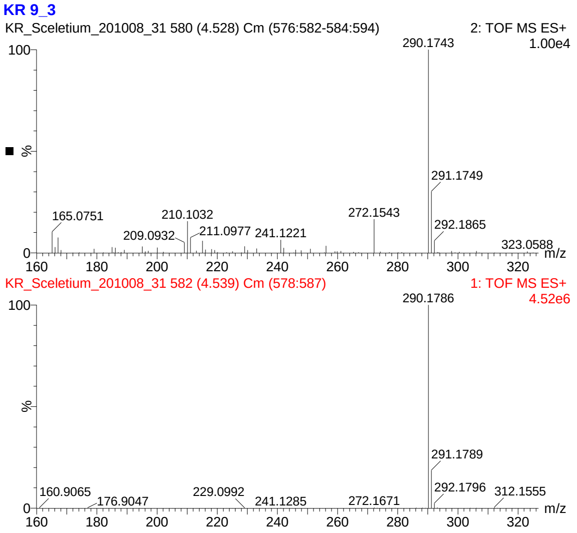

Spectral data for 6-epimesembranol

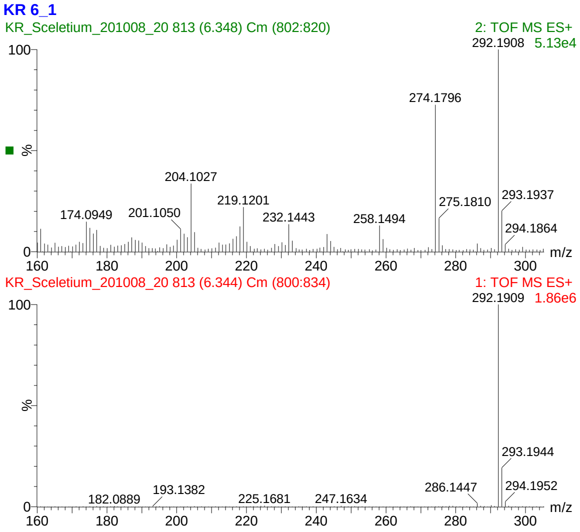

Spectral data for dihydrojoubertiamine

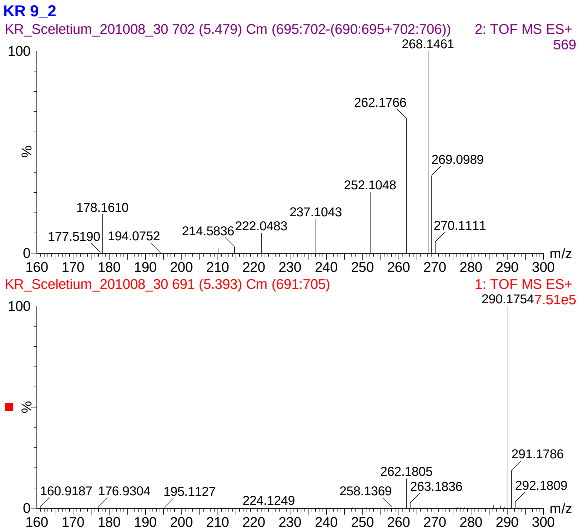

Spectral data for O-methydehydroljoubertiamine

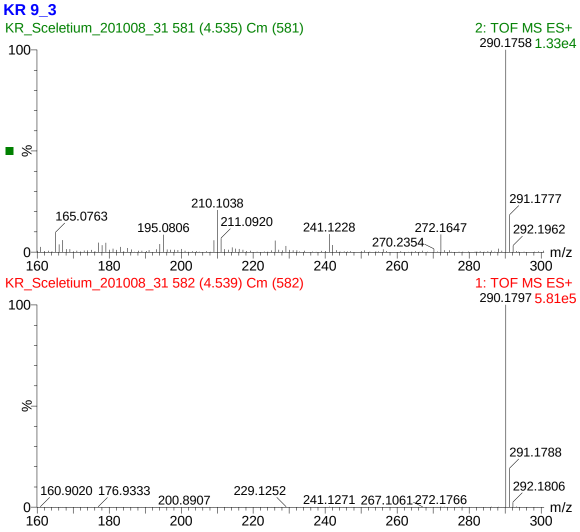

Spectral data for 4-(3,4-dimethyoxyphenyl)-4-[2-acetylmethlamino)ethyl]cyclohexanone

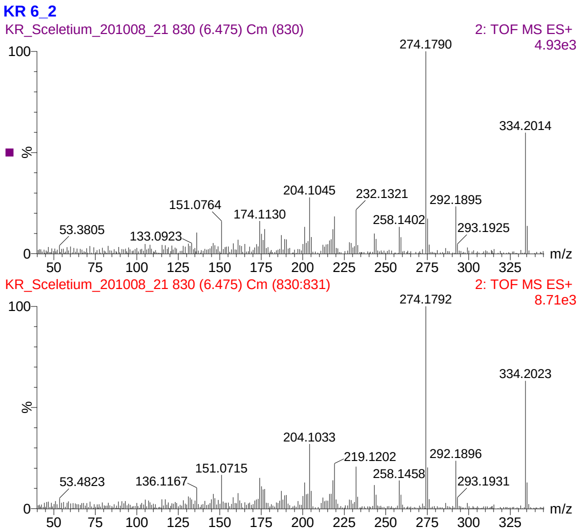

Spectral data for sceletium alkaloid A4

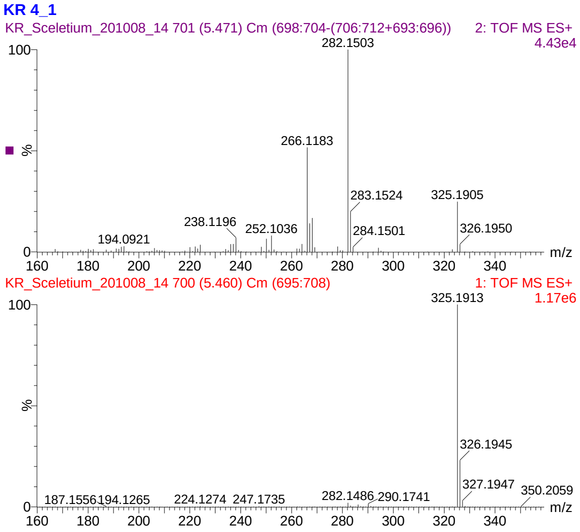

Supplement: Supplementary file 2 [file Data_Sheet_2.PDF]
