# Supplementary material for: Mass Spectrometry Metabolomics and Feature-Based Molecular Networking Reveals Population-Specific Chemistry in Some Species of the Sceletium Genus
Source: Front Nutr. 2022 Mar 29;9:819753. doi: 10.3389/fnut.2022.819753 (PMC9001948; doi:10.3389/fnut.2022.819753)
Supplement: Supplementary file 3 [file Data_Sheet_3.PDF]

Supplementary C

Model validation

PLS-DA cross-validation details for all population (n=12) comparisons:

**Measure**	**1 comps**	**2 comps**	**3 comps**
Accuracy	0.1087	0.21739	0.44565
R2	0.35673	0.48452	0.55292
Q2	0.27219	0.37704	0.40083

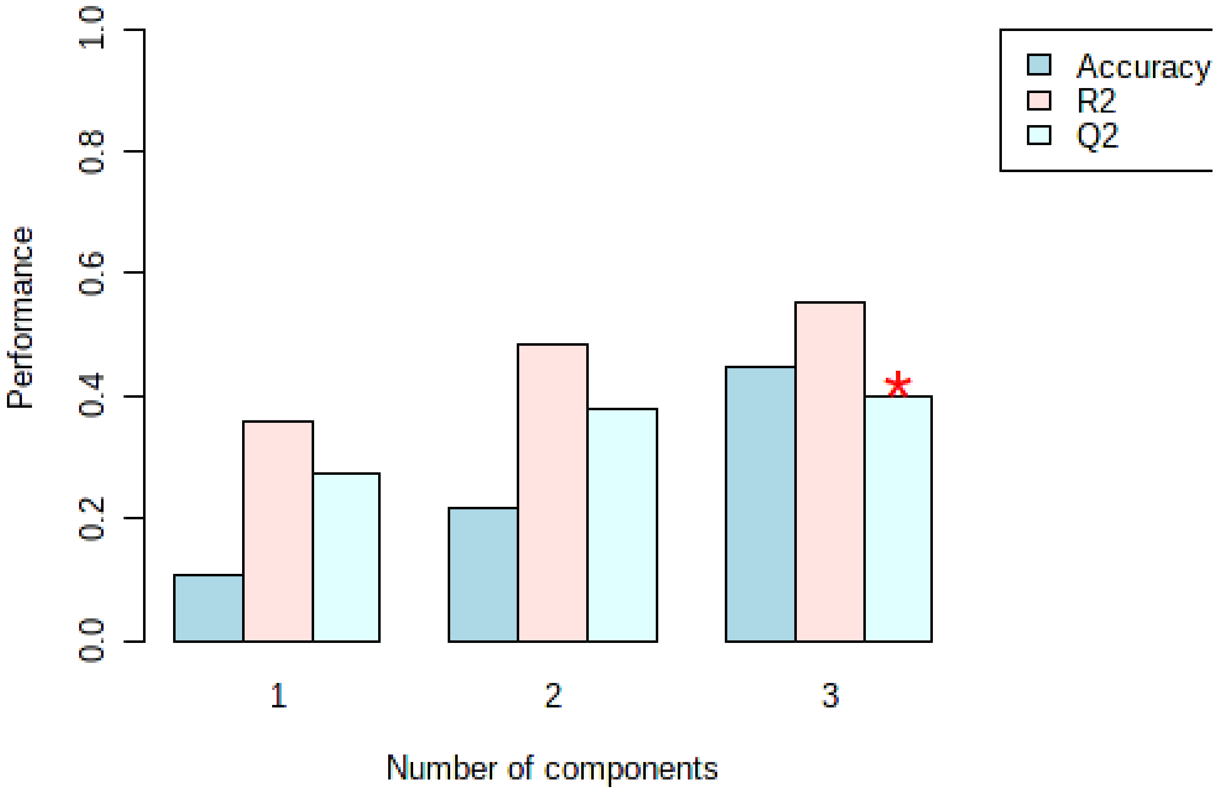

Figure 12: PLS-DA classification using different number of components. The red star indicates the best classifier. Cross validation performed on data pertaining to comparison of all twelve populations of *Sceletium* (including the four different species and remaining *S. tortuosum* populations)

PLS-DA cross-validation details for *Sceletium tortuosum* population comparisons:

Measure	1 comps	2 comps	3 comps
Accuracy	0.16129	0.46774	0.56452
R2	0.71322	0.78966	0.83779
Q2	0.64245	0.71451	0.76783

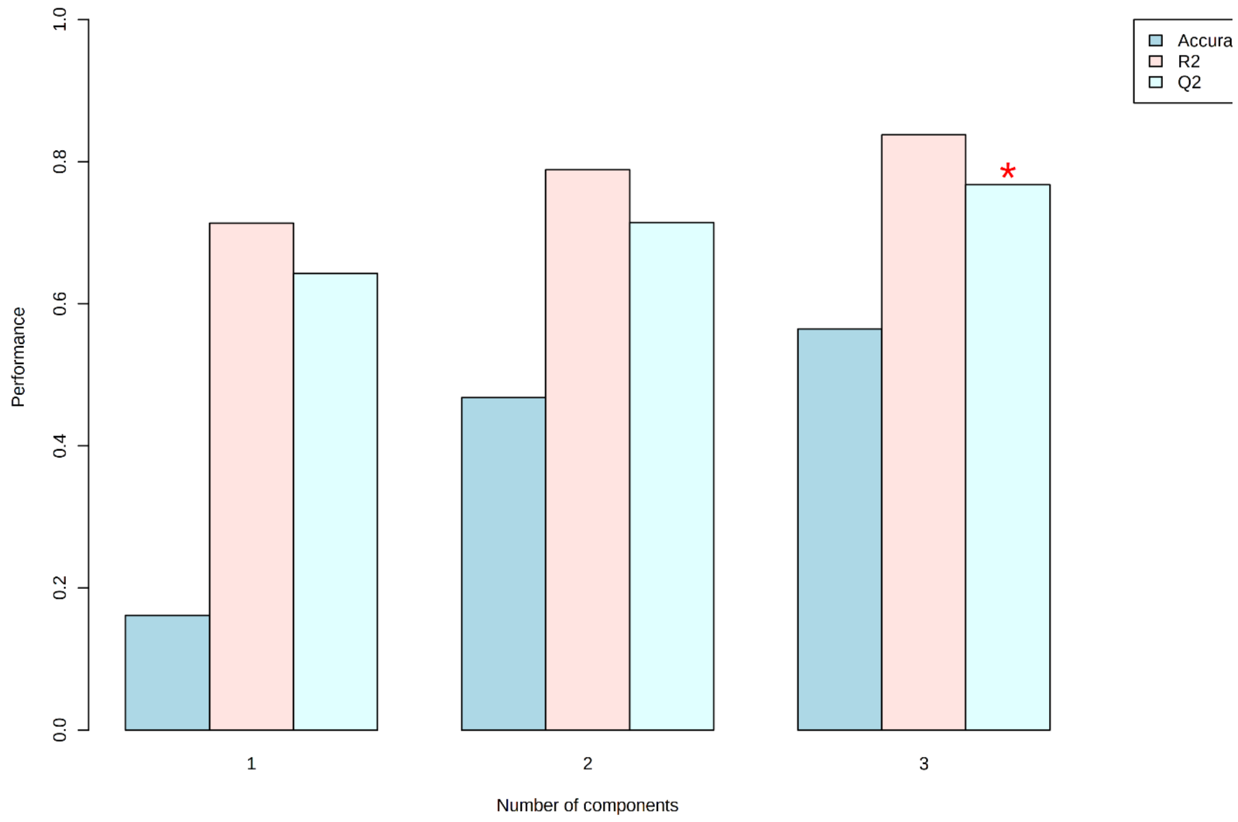

Figure 12: PLS-DA classification using different number of components. The red star indicates the best classifier. Cross validation performed on data pertaining to comparison of populations of *Sceletium tortuosum* collected from different localities (n=9 populations)

PLS-DA cross-validation details for different *Sceletium* species (four species) population comparisons:

Measure	1 comps	2 comps	3 comps	4 comps	5 comps
Accuracy	0.24324	0.78378	0.97297	1.0	1.0
R2	0.64581	0.91678	0.94608	0.96935	0.97838
Q2	0.57916	0.88806	0.91537	0.93542	0.93551

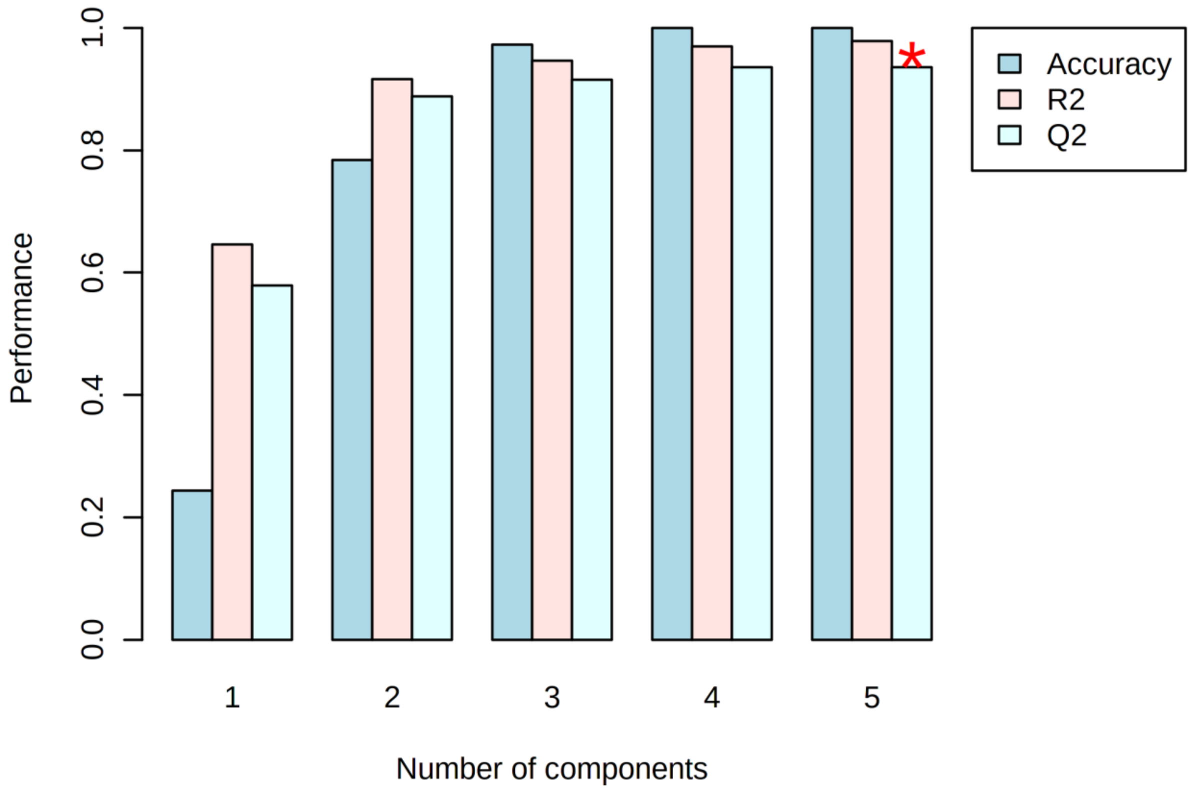

Figure 12: PLS-DA classification using different number of components. The red star indicates the best classifier. Cross validation performed on data pertaining to comparison of 4 species of *Sceletium* (n=10 individuals per population)
