# Supplementary material for: Mass Spectrometry Metabolomics and Feature-Based Molecular Networking Reveals Population-Specific Chemistry in Some Species of the Sceletium Genus
Source: Front Nutr. 2022 Mar 29;9:819753. doi: 10.3389/fnut.2022.819753 (PMC9001948; doi:10.3389/fnut.2022.819753)
Supplement: Supplementary file 4 [file Data_Sheet_4.PDF]

Supplementary D

In silico model validation data

Macromolecule	PDB Code	Positive control	RMSD
**Serotonin uptake inhibitor (5-HT)**	5I75	Citalopram	0.601
**Gamma aminobutyric acid subtype A (GABA-A)**	6D6T	Flumazenil	0.451
**Acetylcholinesterase (AChE)**	1QTI	Galantamine	0.181

Table 6: Validation of molecular docking ligand sites. An RMSD value for the reference ligand <1.000 indicates an appropriate model
